# The epidemiology and burden of smoking in countries of the Association of Southeast Asian Nations (ASEAN), 1990–2021: findings from the Global Burden of Disease Study 2021

**DOI:** 10.1016/S2468-2667(24)00326-8

**Published:** 2025-05-27

**Authors:** GBD 2021 ASEAN Tobacco Collaborators, Xiaochen Dai, Marie Ng, Gabriela Fernanda Gil, Brooks W Morgan, Jason A Anderson, Qorinah Estiningtyas Sakilah Adnani, Budi Aji, Syed Mohamed Aljunid, Gianna Gayle Herrera Amul, Sumadi Lukman Anwar, Geminn Louis Carace Apostol, Kurnia Dwi Artanti, Sarunya Benjakul, Amiel Nazer C Bermudez, Bryan Chong, Dinh-Toi Chu, Thanh Chi Do, Ferry Efendi, Diyan Ermawan Effendi, Nelsensius Klau Fauk, Arief Hargono, Eka Mishbahatul Marah Has, Hong-Han Huynh, Endang Indriasih, Muhammad Iqhrammullah, Ammar Abdulrahman Jairoun, Kehinde Kazeem Kanmodi, Helda Khusun, Maria Dyah Kurniasari, Dian Kusuma, Tri Laksono, Nhi Huu Hanh Le, Thao Thi Thu Le, Stefan Ma, Roy Rillera Marzo, Mustapha Mohammed, Christopher J L Murray, Gustavo G Nascimento, Phat Tuan Nguyen, Van Thanh Nguyen, Dina Nur Anggraini Ningrum, Efaq Ali Noman, Erin M O'Connell, Sok King Ong, Bedanta Roy, Sher Zaman Safi, Made Ary Sarasmita, Siddharthan Selvaraj, Sunil Shrestha, Solikhah Solikhah, Chandrashekhar T Sreeramareddy, Yen Lian Tan, Ingan Ukur Tarigan, Jansje Henny Vera Ticoalu, Thien Tan Tri Tai Truyen, Narayanaswamy Venketasubramanian, Maniphanh Vongphosy, Tati Suryati Suryati Warouw, Angga Wilandika, Siti Rosemawati Yussof, Simon I Hay, Emmanuela Gakidou

## Abstract

**Background:**

Tobacco smoking has long been a regional health priority for the Association of Southeast Asian Nations (ASEAN). Despite decades of commitment to implementing tobacco control measures, the ASEAN region continues to face substantial challenges in reversing the epidemic. We aimed to analyse longitudinal data on smoking prevalence and attributable disease burden to understand the trajectory of the smoking epidemic, inform priority setting, and enable effective policy planning.

**Methods:**

We used data from the Global Burden of Diseases, Injuries, and Risk Factors Study (GBD) 2021 to evaluate the prevalence of tobacco smoking and its attributable disease burden in the ten ASEAN member states by age and sex, from 1990 to 2021. Current smoking prevalence was estimated using spatiotemporal Gaussian process regression models, which synthesised data from 159 distinctive data sources specific to the ASEAN region in addition to 2646 data sources from other GBD countries. Dose–response risks for 36 health outcomes were derived using the latest burden of proof approach. Population attributable fractions were subsequently calculated and applied to determine the burden in terms of mortality, years of life lost, years lived with disability, and disability-adjusted life-years (DALYs) attributable to tobacco smoking in these countries.

**Findings:**

In 2021, there were approximately 137 million (95% uncertainty interval 134–139) current smokers aged 15 years and older in the ASEAN region, with an estimated age-standardised prevalence of 48·4% (47·5–49·2) among males and 4·47% (4·09–4·92) among females. Tobacco smoking accounted for 10·8% (8·86–12·9) of all-cause mortality across the region. The total number of deaths and DALYs attributed to smoking were 526 000 deaths (433 000–622 000) and 15·7 million (12·9–18·5) DALYs. Death rates varied considerably across the region, especially among males, ranging from 68·9 (55·8–84·2) per 100 000 males in Singapore to 364 (279–463) per 100 000 males in Cambodia. Although smoking prevalence declined substantially in most ASEAN countries between 1990 and 2021, the absolute number of smokers increased by 63·3% (59·0–67·8), and the number of smokers aged 10 years and older increased by 53·0 million (50·2–56·2).

**Interpretation:**

Tobacco smoking remains a persistent public health threat in the ASEAN region. Considerable disparities exist across the region: while some countries have made remarkable progress in tobacco control, others lag behind. As a modifiable risk factor heavily influenced by commercial determinants, smoking can be controlled through effective policy changes. As a geopolitical and economic collaboration network, ASEAN countries must work together to overcome barriers hindering anti-tobacco efforts and collectively devise strategies to strengthen tobacco control across the region.

**Funding:**

Bloomberg Philanthropies and the Bill & Melinda Gates Foundation.

## Introduction

Tobacco smoking is a major public health issue across the member states of the Association of Southeast Asian Nations (ASEANs). Consisting of high-income countries (Brunei and Singapore), and low-income and middle-income countries (Cambodia, Indonesia, Laos, Myanmar, Malaysia, Philippines, Thailand, and Viet Nam), the ASEAN region is home to 678 million people, which accounts for roughly 8·6% of the world population.^1^ Compared with other regions, the burden of smoking in the ASEAN is disproportionately high.^2^ In 2019, it was estimated that 134 million smokers, roughly 12% of the total global smokers older than 15 years, resided in the region.^3^, ^4^ Six ASEAN countries—Cambodia, Indonesia, Laos, Malaysia, Myanmar, and the Philippines—rank among the top nations of smoking prevalence in men, with estimates exceeding 40%.^3^, ^4^ Smoking prevalence among youth and adolescents (aged 13–24 years) was high.^5^, ^6^ Additionally, tobacco consumption volume in the region has been estimated to exceed the global average.^7^, ^8^ Subsequent to these high levels of exposure, smoking directly contributed to more than 600 000 deaths in the region in 2019.^9^ For the ASEAN, tackling the tobacco epidemic is of utmost importance.

**Research in context**

**Evidence before this study**

We conducted a systematic literature search of Ovid MEDLINE and PubMed for articles published from database inception to Aug 28, 2024, using the terms “smoking” AND (“prevalence” OR “epidemiology”) AND “disease burden” AND (“ASEAN” OR “Southeast Asia”) (and synonyms for each) with no language or year restrictions. Most of the articles focused on southeast Asia as a region, with none specifically addressing ASEAN. Additionally, the published literature typically presented findings from single-country studies, using data from cross-sectional national or school-based surveys or other national statistics databases. We also examined the grey literature. The Southeast Asia Tobacco Control Alliance publishes regular updates of the ASEAN Tobacco Control Atlas, which provides an overview of the latest epidemic status and relevant policies. Similarly, the ASEAN Secretariats intermittently release tobacco-control reports. However, a temporally and geographically consistent assessment of smoking epidemiology across the ten ASEAN nations is largely lacking.

**Added value of this study**

To our knowledge, this is the first comprehensive study on smoking prevalence and its associated disease burden within ASEAN. As opposed to a conventional geographical definition of region, we focused on ASEAN, a geopolitical and economic collaboration network that plays a pivotal part in shaping trade and economic integration within the region. Understanding the smoking epidemiological landscape within this regional context is important, given the substantial influence of commercial determinants on smoking. Leveraging the results from the Global Burden of Diseases, Injuries, and Risk Factors Study 2021, we examined the historical path and current landscape of smoking prevalence and the attributable burden across all ASEAN member states. Through systematic curation and synthesis of data from multiple sources, geographically and temporally comparable estimates were derived to enable the assessment of smoking trends and burden at both country and regional levels. The findings highlight areas that target country-specific strategies and opportunities for further regional collaboration.

**Implications of all the available evidence**

Smoking prevalence trends and attributable disease burdens differ substantially within the ASEAN region. Although many countries have made inroads in reducing smoking prevalence and burden, progress has been slow overall and has even reversed in some countries. The divergence in country outcomes highlights the unevenness of tobacco-control efforts across the region. As the tobacco industry continues to expand its foothold in the region, implementing tobacco-control measures has become increasingly challenging. Anti-tobacco efforts demand not only stronger political will but also concerted regional strategies to counter the industry's prevailing influence.

The tobacco problem across ASEAN countries is interconnected through multiple dimensions. Free trade and economic cooperation within ASEAN allow the tobacco industry to expand its reach across borders, undermining national control efforts.^10^ Cross-border advertising, illicit trade, and smuggling further complicate enforcement.^11^, ^12^ Additionally, ASEAN countries face common barriers such as economic reliance on tobacco farming,^13^, ^14^ political lobbying by the industry,^15^, ^16^ and varying levels of public health infrastructure, making coordinated regional action essential for effective control.^17^, ^18^

To support the effective implementation of tobacco-control policies, reliable and robust data monitoring is essential. Although numerous studies have investigated the status of the tobacco epidemic in the region, most focused on individual countries or reported cross-sectional surveys within a limited timeframe.^19^, ^20^, ^21^, ^22^, ^23^, ^24^, ^25^ There is a notable absence of a comprehensive and dependable source of information that allows for assessment of smoking prevalence and smoking-associated disease burden within and across the ASEAN region over time. This study seeks to address this gap by providing an updated synthesis of historical and current data from diverse sources to generate comparable country-level time-series estimates for smoking prevalence and disease burden attributable to smoking across ASEAN countries.

This manuscript was produced as part of the Global Burden of Diseases, Injuries, and Risk Factors Study (GBD) Collaborator Network and in accordance with the GBD Protocol.

## Methods

### Overview

As part of GBD 2021, we estimated the prevalence and the burden of disease attributable to tobacco smoking using a comparative risk assessment model across all ten ASEAN member states, stratified by age group (5-year intervals) and sex. In line with the GBD 2021 framework, we present estimates covering the period from 1990 to 2021. Although exposure to second-hand smoke, smokeless tobacco, and heated tobacco products add to the disease burden, they were not considered in this analysis. Hereafter, smoking will be used as a synonym for smoked tobacco use in this paper, inclusive of cigarettes, bidis, kreteks, and other products. The sections below describe the key steps in the process. Comprehensive details on each of these analyses can be found in the appendix (pp 25–30). This research complies with the Guidelines for Accurate and Transparent Health Estimates Reporting (GATHER).^26^ Ethical approval was not required for this study as the data used are publicly available, de-identified, and aggregate in nature.

### Prevalence of smoking

We performed a systematic review of surveys to estimate the prevalence of current and former smoking in ASEAN countries. Current smoking was defined as current use of any smoked tobacco product on a daily or occasional basis. Only nationally or subnationally (state or province level) representative surveys were included, whereas non-representative data were excluded. The systematic review captured 159 qualifying surveys from ASEAN countries (appendix p 24). Individual-level microdata or reported tabulations were extracted for individuals aged 10 years and older. Tabulated estimates that relied on samples smaller than ten were discarded to avoid inaccuracies. Quality checks were conducted after data extraction to eliminate inconsistent or implausible data. Details about data sources are provided in the appendix (pp 4–24). Additional information regarding the search strategy, inclusion criteria, and data extraction methodologies is available in previous publications.^5^

To maintain consistency and eliminate compositional bias, we matched all case definitions of current smoking to our reference case definition. We used spatiotemporal Gaussian process regression (ST-GPR) to model prevalence for both current and former smoking.^9^ Among the 1000 draws from the posterior distribution of ST-GPR, the 2·5th and the 97·5th percentile draws were used to construct uncertainty intervals (UIs) around our final estimates. More information on these methodologies and ST-GPR can be found in previous publications.^9^, ^27^

### Exposure distribution

To estimate the deaths and disability-adjusted life-years (DALYs) attributable to smoking, we evaluated the distributions of smoking intensity among current smokers and the number of years since quitting among former smokers. The intensity of smoking was gauged using both daily cigarette-equivalents of tobacco per smoker and lifetime cumulative pack-years. Data sources included self-reported consumption data from household surveys and national tobacco consumption data from Euromonitor, the US Department of Agriculture, and the Food and Agriculture Organization of the UN. Pack-year distributions were derived from simulated individual smoking histories using distributions of daily cigarette-equivalents per smoker and age of smoking initiation, whereas data on age of cessation helped simulate the number of years since quitting. Further details on our modelling approach to these exposure distributions are available in previous publications.^9^

### Dose–response risk curves

In GBD 2021, dose–response risks curves for 36 health outcomes were derived using the latest burden of proof approach. In short, the burden of proof approach leverages the meta-regression–Bayesian, regularised, trimmed method (MR-BRT) to estimate the dose–response risks associated with current smoking for 36 health outcomes.^28^ Similar to a conventional meta-analytic approach, MR-BRT synthesises data from multiple sources to derive an estimation of average risk. However, distinct from conventional methods, MR-BRT incorporates features that allow for flexible modelling of non-linear risk functions. Further, it was designed to better capture different sources of variability and uncertainty, including between-study heterogeneity, uncertainties stemming from small numbers of studies, varying exposure ranges between groups, and biases associated with study designs and features.

Based on an updated systematic literature review in 2022, a total of 793 studies reporting smoking risks on the 36 outcomes among various populations were included, whereas studies among people with pre-existing conditions were excluded.^29^ For former smokers, we estimated risk reduction at different years since cessation. To standardise the risks at the point of cessation, we used the exposure-weighted risk integral from current smokers, adjusting for age, sex, location, and year. More details about the methods can be found in previous publications and in the appendix (p 28).^7^, ^9^, ^29^ Data sources used in this analysis can be found in the appendix (pp 4–24).

### Population attributable fractions

Details on the calculation of population attributable fractions (PAFs) can be found in the appendix (p 30). Deaths and DALYs attributable to smoking were calculated by applying a given PAF to the corresponding estimate of cause-specific deaths and DALYs by year, location, age group, and sex. All analyses were performed using R (version 3.6–4.1) and Python (version 3.3).

### Role of the funding source

The funders of this study had no role in study design, data collection, data analysis, data interpretation, or the writing of the report.

## Results

**Figure 1:**
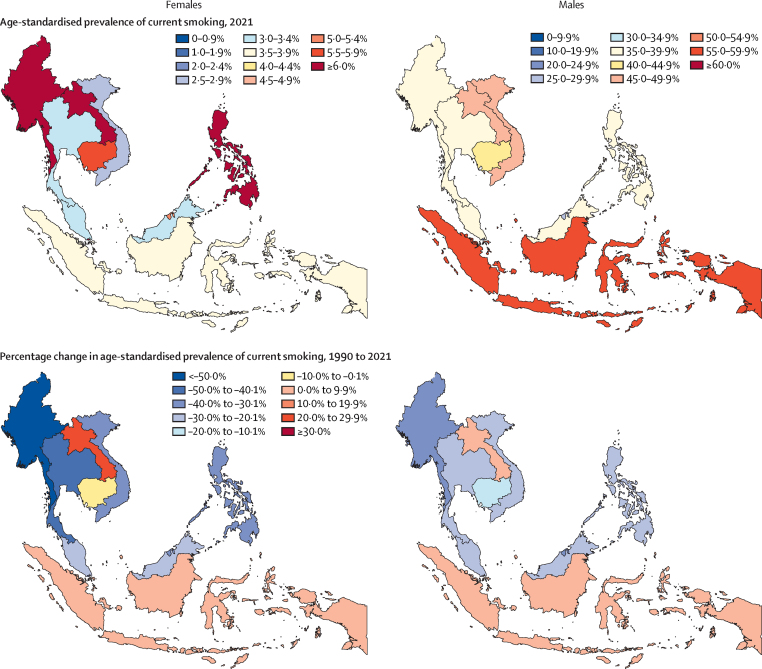
Age-standardised prevalence of smoking in 2021 and percentage change in age-standardised prevalence of smoking between 1990 and 2021 among females and males aged 15 years and older, by ASEAN country ASEAN=The Association of Southeast Asian Nations.

In 2021, the age-standardised prevalence of smoking among males aged 15 years and older in the ASEAN was estimated to be 48·4% (95% UI 47·5–49·2; table 1). Nearly all member states, with the exception of Brunei and Singapore, had an estimated prevalence in males approaching or exceeding 40% (table 1, figure 1). Indonesia had the highest prevalence of smoking in males, at 57·8% (56·2–59·4). The estimated regional prevalence of smoking among females aged 15 years and older was markedly lower, at 4·47% (4·09–4·92). Myanmar (8·01% [6·63–9·46]) and the Philippines (7·91% [6·76–9·25]) had the highest prevalence of smoking in females. Although Singapore had the lowest smoking prevalence among males (20·2% [18·4–22·3]), smoking prevalence among females exceeded 6% and ranked the fourth highest in the region.

**Table 1:** Age-standardised prevalence of any current smoking use and percentage change in age-standardised prevalence of any current smoking use between 1990 and 2021 among individuals aged 15 years and older, by location and sex

	**Females**			**Males**		
	Age-standardised prevalence		Percentage change in prevalence	Age-standardised prevalence		Percentage change in prevalence
	1990	2021	1990–2021	1990	2021	1990–2021
ASEAN region	6·48% (5·94 to 6·99)	4·47% (4·09 to 4·92)	−30·9% (−39·0 to −21·5)	55·3% (54·3 to 56·3)	48·4% (47·5 to 49·2)	−12·5% (−14·6 to −10·3)
Brunei	8·06% (6·42 to 9·99)	5·05% (3·94 to 6·39)	−36·4% (−54·0 to −13·8)	43·5% (40·7 to 46·4)	27·1% (24·4 to 29·6)	−37·7% (−44·5 to −30·4)
Cambodia	5·79% (4·72 to 7·05)	5·53% (4·53 to 6·67)	−3·47% (−29·9 to 26·0)	49·0% (46·8 to 51·3)	40·5% (38·6 to 42·6)	−17·3% (−22·9 to −11·4)
Indonesia	3·40% (2·80 to 4·08)	3·54% (2·92 to 4·36)	5·08% (−22·0 to 39·0)	55·3% (53·6 to 57·0)	57·8% (56·2 to 59·4)	4·54% (0·427 to 8·55)
Laos	5·47% (4·25 to 6·94)	6·82% (5·64 to 8·28)	26·8% (−7·11 to 73·5)	45·0% (42·2 to 47·8)	48·6% (46·1 to 51·1)	8·08% (−0·899 to 17·5)
Malaysia	4·19% (3·27 to 5·26)	3·06% (2·46 to 3·80)	−25·8% (−47·9 to 1·30)	50·3% (47·3 to 53·3)	39·9% (37·3 to 42·4)	−20·7% (−27·7 to −13·6)
Myanmar	16·2% (13·6 to 19·0)	8·01% (6·63 to 9·46)	−50·2% (−61·1 to −38·0)	58·7% (56·1 to 61·6)	39·6% (37·6 to 41·8)	−32·5% (−37·1 to −27·9)
Philippines	13·2% (10·9 to 15·7)	7·91% (6·76 to 9·25)	−39·4% (−52·9 to −23·5)	56·9% (54·3 to 59·6)	39·9% (37·9 to 41·9)	−29·9% (−34·4 to −24·9)
Singapore	9·84% (7·77 to 12·5)	6·56% (5·20 to 8·19)	−32·3% (−51·5 to −7·00)	28·6% (26·3 to 31·3)	20·2% (18·4 to 22·3)	−29·2% (−37·9 to −19·8)
Thailand	6·30% (5·22 to 7·55)	3·36% (2·70 to 4·09)	−46·2% (−59·6 to −29·4)	52·5% (50·0 to 55·1)	39·9% (38·0 to 41·8)	−24·0% (−29·2 to −18·5)
Viet Nam	3·97% (3·24 to 4·76)	2·57% (2·04 to 3·20)	−34·5% (−52·6 to −13·7)	59·0% (57·0 to 61·1)	46·9% (45·0 to 48·9)	−20·5% (−24·8 to −15·9)

ASEAN=The Association of Southeast Asian Nations. Data are presented to three significant figures.

**Figure 2:**
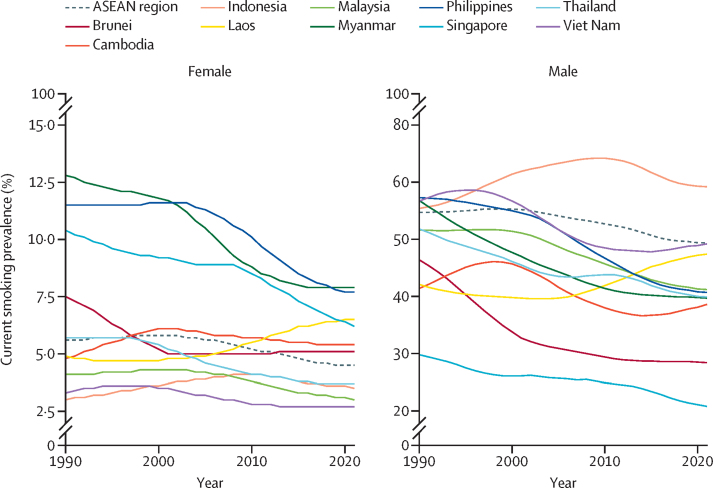
Changes in current smoking prevalence in females and males, by ASEAN country, 1990–2021 Note that the y axes are at different scales in the female and male graphs and that the axes have been broken at different points, to allow better visualisation of the data. ASEAN=The Association of Southeast Asian Nations.

Between 1990 and 2021, substantial reductions in male smoking prevalence were achieved in eight ASEAN countries: Brunei, Cambodia, Malaysia, Myanmar, the Philippines, Singapore, Thailand, and Viet Nam (figure 2, table 1). The percentage of decline in prevalence ranged from 17·3% (95% UI 11·4 to 22·9) in Cambodia to 37·7% (30·4 to 44·5) in Brunei. Conversely, Indonesia experienced an increase in smoking prevalence among males of 4·54% (0·427 to 8·55). The percentage change in smoking prevalence among males in Laos was 8·08% (–0·899 to 17·5); however, the uncertainty interval for that change encompassed 0%. As for smoking prevalence among females, six of the ten ASEAN countries observed substantially reductions. In Myanmar, female smoking prevalence dropped by 50·2% (38·0 to 61·1). Thailand also observed a substantial percentage reduction of 46·2% (29·4 to 59·6) over the same period. Brunei, the Philippines, Singapore, and Viet Nam also observed declines of more than 30% in prevalence. However, female smoking prevalence in Cambodia, Indonesia, Laos, and Malaysia remained stagnant, with no statistically significant variations identified during this timeframe. An examination of the annualised rate of change (appendix p 32) shows that the region as a whole observed rapid increases in the number and prevalence of female smokers between 2000 and 2010; however, the rates of growth have slowed down substantially between 2016 and 2021.

The prevalence of smoking among youth aged 10–14 years varied considerably within the region. In 2021, the prevalence of smoking among male children and adolescents ranged from 2·80% (95% UI 1·78–4·25) in Singapore to 20·4% (15·4–25·9) in Malaysia (table 2). The prevalence of smoking among female children and adolescents was substantially lower. The highest prevalence was observed in the Philippines at 7·03% (3·70–12·2), and the lowest prevalence was observed in Indonesia, at 1·31% (0·504–2·65). Compared with 1990 estimates, although the prevalence among female children and adolescents remained stable in all countries, important increases were observed in smoking prevalence among male children and adolescents in several countries. For instance, Cambodia witnessed a substantial rise of 116% (13·8–274) in male youth smoking prevalence, soaring from 1·76% (1·08–2·64) in 1990 to 3·62% (2·33–5·43) in 2021. Indonesia had a substantial surge of 79·1% (0·429–210), climbing from 6·27% (4·05–9·16) in 1990 to 10·7% (7·37–14·9) in 2021. Two countries, the Philippines and Singapore, achieved a substantial reduction in smoking prevalence among male children and adolescents between 1990 and 2021, with declines of 41·4% (11·6–63·4) in the Philippines and 55·0% (23·0–76·2) in Singapore.

**Table 2:** Age-standardised prevalence of youth smokers and percentage change in age-standardised prevalence of any current smoking use between 1990 and 2021 among individuals aged 10–14 years, by location and sex

	**Females**			**Males**		
	Age-standardised prevalence		Percentage change in prevalence	Age-standardised prevalence		Percentage change in prevalence
	1990	2021	1990–2021	1990	2021	1990–2021
ASEAN region	3·95% (2·69 to 5·77)	3·01% (2·10 to 4·12)	−21·1% (−54·9 to 29·7)	10·9% (9·09 to 12·8)	11·1% (9·34 to 13·2)	2·51% (−19·2 to 29·6)
Brunei	2·35% (0·847 to 4·95)	1·96% (0·739 to 4·14)	0·79% (−73·4 to 162)	5·70% (3·54 to 8·63)	4·97% (3·26 to 7·26)	−8·27% (−51·4 to 63·7)
Cambodia	1·14% (0·421 to 2·52)	3·17% (1·44 to 5·87)	245% (−9·16 to 874)	1·76% (1·08 to 2·64)	3·62% (2·33 to 5·43)	116% (13·8 to 274)
Indonesia	1·16% (0·467 to 2·57)	1·31% (0·504 to 2·65)	34·0% (−66·4 to 232)	6·27% (4·05 to 9·16)	10·7% (7·37 to 14·9)	79·1% (0·0429 to 210)
Laos	2·36% (0·927 to 5·24)	2·42% (0·99 to 4·91)	23·2% (−67·5 to 228)	8·32% (5·37 to 12·3)	8·77% (6·02 to 12·0)	10·3% (−38·1 to 80·5)
Malaysia	4·38% (1·77 to 9·18)	4·03% (2·04 to 7·63)	10·5% (−67·4 to 176)	25·0% (17·8 to 33·7)	20·4% (15·4 to 25·9)	−16·0% (−46·2 to 22·4)
Myanmar	5·90% (2·45 to 11·9)	3·53% (1·49 to 6·92)	−28·9% (−81·4 to 81·2)	19·0% (13·2 to 26·1)	15·9% (12·0 to 21·1)	−13·2% (−46·7 to 38·2)
Philippines	13·9% (6·75 to 25·1)	7·03% (3·70 to 12·2)	−43·8% (−78·9 to 23·2)	25·5% (18·5 to 34·3)	14·5% (10·8 to 19·0)	−41·4% (−63·4 to −11·6)
Singapore	5·36% (2·34 to 10·4)	2·55% (0·98 to 5·68)	−45·1% (−84·6 to 57·2)	6·53% (4·18 to 9·76)	2·80% (1·78 to 4·25)	−55·0% (−76·2 to −23·0)
Thailand	4·08% (1·99 to 7·51)	3·19% (1·40 to 6·32)	−10·9% (−73·0 to 118)	11·6% (7·84 to 16·4)	10·8% (7·38 to 15·2)	−3·18% (−44·2 to 59·1)
Viet Nam	1·22% (0·456 to 2·66)	1·36% (0·509 to 2·94)	31·7 (−64·4 to 249)	2·98% (1·88 to 4·57)	3·46% (2·16 to 5·26)	21·7 (−38·3 to 114)

ASEAN=The Association of Southeast Asian Nations. Data are presented to three significant figures.

The appendix (p 34) shows the total number of smokers aged 15 years and older by country. For 2021, we estimated a total of 137 million (95% UI 134–139) current smokers in the ASEAN region, of whom 125 million (123–127) were male. Due in part to its large population, Indonesia accounted for over half of the current male smoking population, with 63·2 million (61·4–64·9) male smokers. Viet Nam (18·3 million [17·6–19·1]) and the Philippines (16·2 million [15·4–17·1]) also had relatively high numbers of male current smokers. The number of female current smokers was relatively high in two countries: Indonesia, with 3·72 million (3·02–4·63), and the Philippines, with 3·02 million (2·56–3·53).

Despite the reduction in smoking prevalence in most ASEAN countries, the absolute number of smokers increased by 63·3% (59·0–67·8) between 1990 and 2021, and the region gained 53·0 million (95% UI 50·2–56·2) smokers. The largest absolute increase was observed in Indonesia, with an addition of 33·2 million (31·0–35·5) smokers. In terms of relative percentage change, Laos observed the largest difference in the number of smokers during the period, an increase of 160% (137–184), followed by Cambodia with an increase of 110% (93·0–128). Myanmar and Thailand recorded the lowest relative increase of less than 10%.

**Figure 3:**
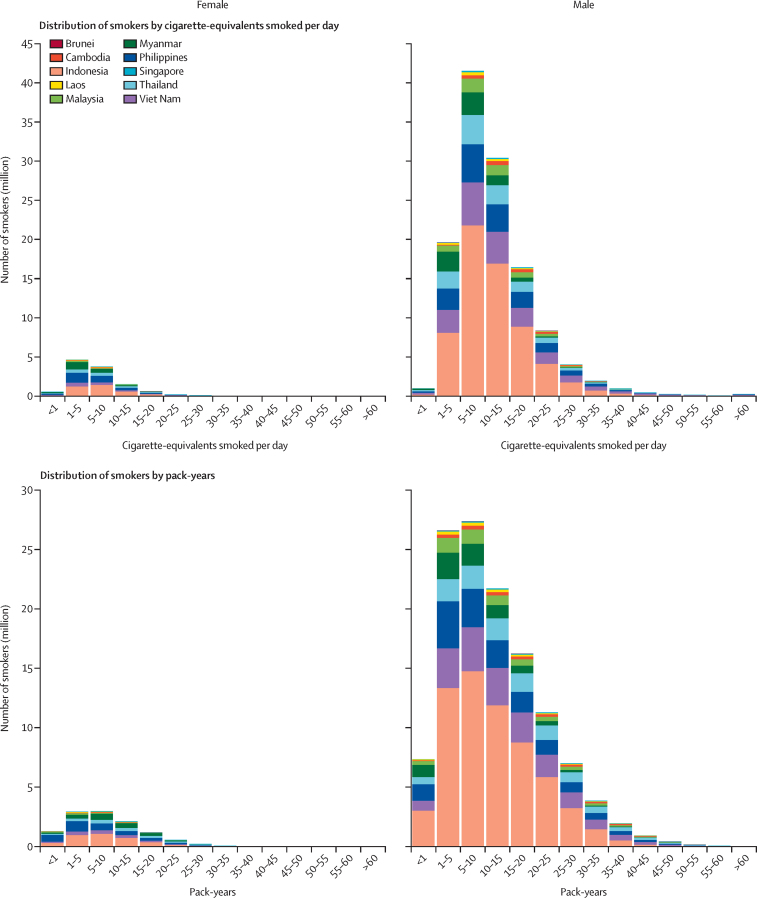
Distribution of female and male current smokers aged 15 years and older according to cigarette-equivalents smoked per day and pack-years, by ASEAN country, 2021 ASEAN=The Association of Southeast Asian Nations.

In 2021, the total number of cigarette-equivalent units of tobacco smoked in the ASEAN region was estimated to be 566 billion (95% UI 539–596; appendix p 35). Indonesia accounted for nearly half of the consumption in the region (280 billion units [257–304]). Indonesia also observed the highest consumption per smoker, which was estimated to be 4190 cigarette-equivalents (3860–4530) per smoker aged 15 years and older per year. The lowest consumption was observed in Myanmar, which was estimated to be 2560 cigarette-equivalents (2170–3050) per smoker per year. Regionwide, among male smokers aged 15 years and older, 16·4% (20·6 million of 125 million male smokers) consumed less than five cigarette-equivalents per day, 33·1% (41·5 million) consumed five to ten cigarette-equivalents per day, 24·2% (30·4 million) consumed 10–15 cigarette-equivalents per day, and 26·3% (33·1 million) consumed over 15 cigarette-equivalents per day (figure 3). Among female smokers aged 15 years and older, 45·1% (5·22 million of 11·6 million female smokers) consumed less than five cigarette-equivalents per day, 32·7% (3·79 million) consumed five to ten cigarette-equivalents per day, and 22·2% (2·57 million) consumed over ten cigarette-equivalents per day.

In 2021, 10·8% (95% UI 8·86–12·9) of all-cause mortality was attributable to smoking across ASEAN, resulting in 526 000 (433 000–622 000) deaths and 15·7 million (12·9–18·5) DALYs (appendix pp 36–39). Males bore a disproportionate share of the burden, accounting for 90·8% (477 000 of 526 000) of the total smoking-attributable deaths. Years of life lost (YLLs) due to smoking amounted to 14·0 million (11·5–16·5), and the number of years lived with disability (YLDs) was estimated to be 1·68 million (1·16–2·26). This translated to a YLL-to-YLD ratio of 8·32 (6·48–11·2), which indicates that the burden of premature mortality associated with smoking is higher than the burden of health loss due to the smoking-attributable morbidities. However, YLL-to-YLD ratios diverged markedly across countries. High ratios were observed in Laos (10·2 [7·38–14·8] among males and 5·74 [4·27–8·31] among females) and Myanmar (10·4 [7·86–14·7] among males and 6·50 [4·86–9·21] among females). In contrast, low ratios were observed in Singapore (3·09 [2·37–4·28] among males and 1·62 [1·12–2·39] among females) and Brunei (4·30 [3·14–6·08] among males and 3·30 [2·38–4·76] among females; appendix pp 40–41).

The proportion of all-cause mortality attributable to smoking among males was estimated to be 17·4% (95% UI 14·4–20·7) at the regional level, with Cambodia witnessing the highest proportion at 22·8% (18·0–27·7; appendix pp 36–39). For females, the proportion of all-cause mortality attributable to smoking was estimated to be 2·32% (1·78–2·98) at the regional level, with the highest proportion observed in Myanmar at 5·34% (3·73–7·61). The region-wide death rate associated with smoking was estimated to be 281 (232–331) per 100 000 males and 27·9 (21·4–35·4) per 100 000 females. Male death rates exceeded 300 per 100 000 in three countries—Cambodia, Laos, and Viet Nam—with Cambodia observing the highest death rate at 370 (279–460) per 100 000 males. The lowest death rate was observed in Singapore, at 69·4 (56·1–83·4) per 100 000 males. Female death rates ranged from 13·1 (8·70–19·1) per 100 000 in Viet Nam to 75·9 (51·9–108) per 100 000 in Myanmar.

An examination of trends indicated a decline in the proportion of deaths attributable to smoking across the ASEAN region between 1990 and 2021. The proportion of smoking-attributable all-cause mortality decreased by 25·6% (95% UI 16·3–35·6). Notable declines were observed, with the proportion of smoking-attributable all-cause mortality decreasing by 49·0% (42·0–55·3) in the Philippines, 47·3% (37·5–56·6) in Myanmar, and 47·2% (41·2–53·2) in Singapore. However, in absolute terms, the number of deaths attributable to smoking has increased by 231 000 (167 000–303 000) since 1990. The increase was predominantly driven by additional deaths in Indonesia (131 000 [79 900–185 000]), Viet Nam (38 900 [21 200–55 100]), and the Philippines (35 600 [20 900–52 000]) between 1990 and 2021, concurrent with increases in population (appendix p 42).

Specific to smoking-attributable non-communicable disease (NCD) burden, 13·9% (95% UI 11·7–16·1) of all NCD-related mortality across the ASEAN region in 2021 was attributable to smoking as a risk factor, equating to 463 000 (383 000–547 000) NCD-related deaths (appendix p 43). Approximately 13·8 million (11·4–16·2), or 11·2% (9·37–13·2), of NCD-related DALYs were associated with smoking (appendix p 44). Between 1990 and 2021, although the proportion of smoking-attributable NCD mortality decreased by 14·5% (7·80–20·8), the absolute number of NCD deaths attributable to smoking increased by 229 000 (172 000–294 000).

**Figure 4:**
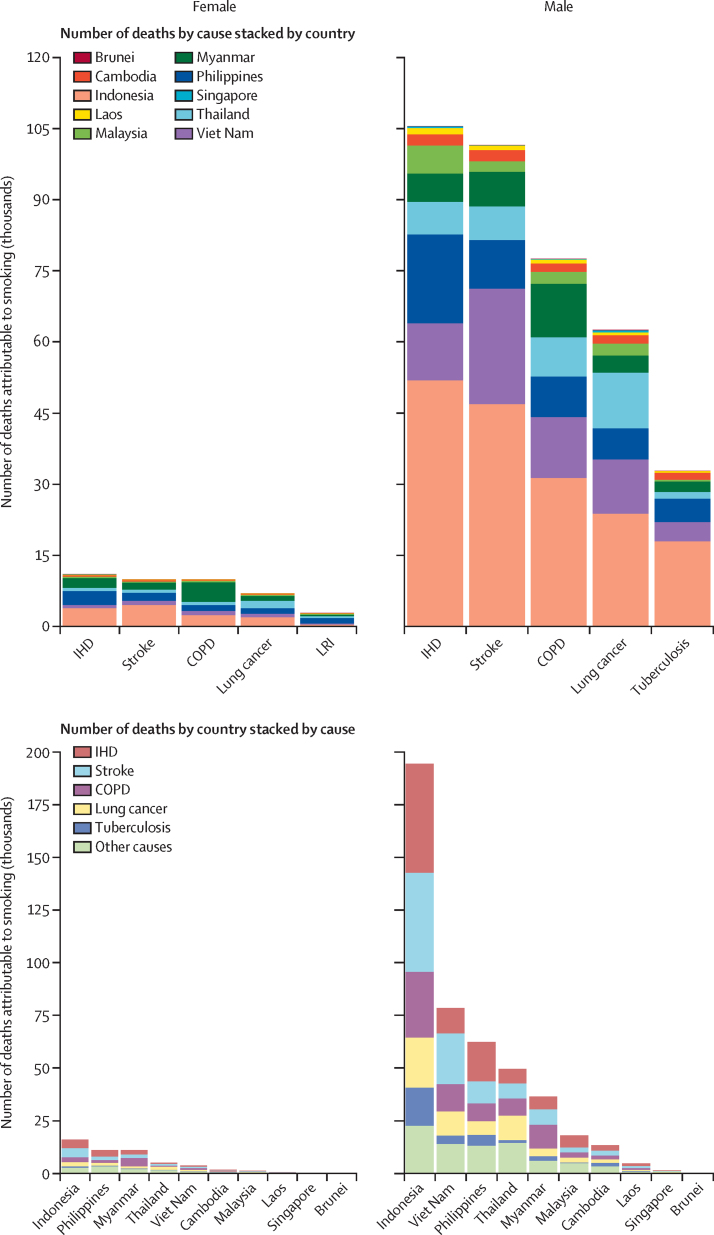
Number of deaths of females and males aged 30 years and older attributable to smoking by top causes and ASEAN country, and by ASEAN country and top causes, 2021 ASEAN=The Association of Southeast Asian Nations. COPD=chronic obstructive pulmonary disease. IHD=ischemic heart disease LRI=lower respiratory infections.

Among the 36 health outcomes associated with smoking, in 2021, ischaemic heart disease accounted for the highest estimated number of deaths in the region (118 000 [95% UI 99 300–139 000]), followed by stroke (113 000 [93 700–133 000]) and chronic obstructive pulmonary disease (89 300 [72 100–107 000]). This ranking was relatively consistent across the ASEAN countries despite slight differences. For instance, in Brunei, Malaysia, Singapore, and Thailand, tracheal, bronchus, and lung cancers superseded stroke as one of the top three causes of smoking-attributable deaths (figure 4; appendix pp 45–46).

## Discussion

In this study, we leveraged the systematic analysis from GBD 2021 to offer a longitudinal perspective on smoking prevalence and its resulting disease burden across the ASEAN region. Our findings revealed an intricate landscape of the progress and setbacks in battling the smoking epidemic in the region between 1990 and 2021. Although some countries have made commendable progress in curbing smoking prevalence, others struggled. Overall, smoking prevalence remains high across the ASEAN region. As of 2021, there were 137 million current smokers aged 15 years and older, with age-standardised prevalence of 48·4% among males and 4·47% among females. Most countries observed a decline in smoking prevalence among males; however, in Laos, no substantial change was observed, and in Indonesia, a substantial increase was found. Although six of the ten member states have achieved substantial declines in female smoking prevalence over the time period studied, no substantial change was observed in the other countries.

Based on the historical trend of smoking prevalence, the tobacco epidemic in each of the ASEAN countries can be categorised into a specific phase. As proposed by Dai and colleagues,^7^ the tobacco epidemic can be roughly delineated into four distinct phases: the incipient phase, characterised by low prevalence; a second phase marked by increasing prevalence without noticeable decline; a third phase in which signs of decline emerge, yet prevalence remains high; and a fourth phase in which prevalence has decreased to relatively low levels. ASEAN member countries are found at varied stages of this tobacco epidemic. For male smoking prevalence, Indonesia is in the second phase, trailing behind the majority. Brunei, Cambodia, Laos, Malaysia, Myanmar, the Philippines, Thailand, and Viet Nam have advanced to the third phase. Only Singapore has reached the fourth phase of the tobacco epidemic among males. For females, Indonesia, Malaysia, Thailand, and Viet Nam remain in the incipient stage; Brunei, Cambodia, and Laos are positioned in the second phase; and Myanmar, the Philippines, and Singapore have advanced to the fourth phase of the epidemic.

The consequences of smoking extend far beyond prevalence. Smoking is a leading risk factor contributing to premature death and disability. In 2021, 526 000 deaths and 15·7 million DALYs were attributable to smoking within the ASEAN region. Moreover, smoking accounted for 11·2% of the burden caused by NCDs. Considerable divergence in the ratios of YLLs to YLDs was observed across the region, signalling the differential impact of smoking on premature mortality and long-term morbidity. This divergence possibly reflects the differences in tobacco consumption patterns, disparities in access to quality health care, and policies related to tobacco control across the region.^30^, ^31^

A troubling finding from our study is the trend of persistent or even rising youth smoking prevalence across much of the ASEAN region. Although smoking rates among youths remained low in most countries, Malaysia emerged as a concerning exception, with over 20% of male children and adolescents aged 10–14 years engaging in smoking. Six of the ten ASEAN member nations have failed to achieve any reduction in youth smoking prevalence since 1990. Meanwhile, Indonesia and Cambodia have witnessed alarming increases in smoking among male children and adolescents. Singapore and the Philippines stand out as the only countries that have successfully reduced smoking among male children and adolescents. It is worth noting that in all ASEAN countries, the legal smoking age is 18 years,^32^ except in Singapore, where it has recently been raised to 20 years.^33^ The high rates of youth smoking below the legal age highlight a concerning gap in the enforcement of tobacco-control policies and the need for enhanced educational efforts. Youth smoking is an early indicator of future adult smoking and subsequent disease burden,^34^, ^35^ and there is an urgent need for more targeted efforts to deter smoking initiation among the younger population.

The high prevalence of tobacco smoking in the ASEAN region has underscored crucial weaknesses in its tobacco-control strategies. Except for Indonesia, all ASEAN nations have ratified the WHO Framework Convention on Tobacco Control, thereby committing to adopting its comprehensive tobacco-control measures, which include actions related to monitoring tobacco use, enacting policy measures, providing cessation programmes, requiring health warnings, instituting advertising bans, and increasing taxation. However, the implementation and enforcement of these measures, as outlined in WHO's MPOWER guidelines, are markedly inconsistent across the region. As revealed in the latest MPOWER report,^36^ although four countries in the ASEAN—Brunei, Cambodia, Laos, and Thailand—have achieved the highest standard by enforcing comprehensive smoking bans in all public spaces, compliance with the smoking bans is merely moderate. Public smoking prohibition coverage is the lowest in Malaysia. In terms of package warnings, all countries except Indonesia and Myanmar have met the highest standard. However, the use of mass media campaigns to disseminate tobacco-control messages is implemented in only half of the ASEAN countries. The enforcement of bans on advertising, promotion, and sponsorship is notably weak in the region, with indirect advertising still permissible in all countries. Regarding taxation, the Philippines is the only country that has raised tobacco taxes adequately over the past decade to make cigarettes less affordable. Although Singapore levies a substantial 66·3% tax on tobacco, this has not resulted in decreased affordability. Cigarettes have become more affordable in four countries over the past decade: Cambodia, Laos, Singapore, and Viet Nam (appendix pp 47–48).

Two structural challenges have been hindering the implementation and enforcement of tobacco-control interventions—namely the absence of government commitment and the strong interference of the tobacco industry.^17^, ^18^, ^37^ The tobacco industry wields considerable influence across the region, using a range of tactics to exert influence on local governments.^12^, ^38^ As one of the fastest growing economies in the world with a young demographic,^39^ the ASEAN region presents substantial business growth opportunities for the tobacco industry.^40^ In addition to the continuous investment of major multinational tobacco companies in the region, local tobacco companies, such as Thailand Tobacco Monopoly (a state-owned enterprise from Thailand) and Gudang Garam (an Indonesian cigarette and kretek company), are actively expanding their presence across the region.^41^ In countries with a strong fiscal reliance on tobacco, the potential revenue loss from reduced tobacco consumption often overshadows long-term public health and economic benefits, undermining the implementation of effective measures such as tobacco taxation.^42^ Ministries of finance in many countries have close ties with the tobacco industry.^43^ For example, the Laos Government signed a 25-year Investment License Agreement with two tobacco companies in 2001, granting the joint venture specific tax exemptions and privileges for tobacco products.^44^ Unless action is taken, the agreement is set to be renewed in 2026.^45^ Studies have also exposed the tobacco industry's effort to manipulate tobacco tax through illicit trade.^43^, ^46^ By supporting the illicit trade market, the tobacco industry attempts to trigger revenue loss from uncollected taxes, causing hesitation in raising tobacco taxes.^1^ However, evidence has shown that with strong governance and an effective tax administration structure, tobacco taxation is effective in reducing tobacco demand and does not drive illicit trade.^47^ It is worth noting that much of the illicit tobacco trade in the ASEAN region occurs among member countries.^11^ In Malaysia, where the incidence of illicit cigarettes is among the highest in the world,^48^ most of these products are smuggled from other ASEAN countries, particularly Viet Nam and Indonesia.^49^ As an economic collaboration network, ASEAN is uniquely positioned to tackle these issues collectively to safeguard the health of its population.

The issue of youth smoking prevention within the ASEAN region has been marked by notable policy delays and gaps. One example is Thailand, which, despite its progressive stance in other areas of tobacco control, did not enact its inaugural tobacco-control legislation and school-based initiatives aimed at youth until 2017.^50^ This delay is crucial given the heightened susceptibility of teenagers to advertising influences, which has been well documented. Studies indicate that the tobacco industry has not only recognised teenagers as a key demographic but has actively engaged in strategies to appeal to this group.^51^, ^52^, ^53^, ^54^ The nature of tobacco industry marketing has evolved, shifting from traditional mass-media campaigns to more covert and insidious below-the-line tactics. These tactics include using social media platforms, which allow for targeted marketing campaigns that can bypass the more visible and regulated forms of advertising.^38^, ^55^ This subtle form of marketing is particularly effective for the younger demographic, which is known to consume a substantial amount of content through these platforms. A study in Myanmar showed that over 90% of high-school students were exposed to tobacco advertising, promotion, and sponsorship (TAPS).^56^ In Indonesia, nearly 30% of students reported online exposure to TAPS on Instagram, and offline exposure through television was as high as 74%.^57^ In Cambodia, at least 54% of adolescents between the ages of 18 and 24 years have been exposed to at least one below-the-line marketing strategy.^58^ In the face of these sophisticated marketing strategies, ASEAN member states have struggled to implement and enforce effective countermeasures. In Singapore, where tobacco-control policies are stricter than in other ASEAN countries, the tobacco industry has adapted its branding strategies by promoting so-called light cigarettes to appeal to the younger generation of smokers.^59^ Policies to thwart the tobacco industry's efforts to entice young people have been inadequate.

Meanwhile, the introduction of non-combustible tobacco products has become a strategy to entice young smokers in the region.^60^, ^61^ Although five of the ten ASEAN countries (Brunei, Cambodia, Laos, Singapore, and Thailand) have banned electronic nicotine delivery systems (ENDS) including vaping, e-cigarettes, and heated tobacco products, other nations have little to no regulatory control in place.^62^ In the Philippines, over 14% of adolescents reported current e-cigarette use in 2019;^63^ a similar prevalence was observed in Malaysia.^64^ This trend is concerning, as evidence suggests that ENDS might act as a gateway to traditional smoking, especially among younger populations.^65^, ^66^, ^67^, ^68^ The lack of comprehensive anti-tobacco marketing laws and comprehensive educational programmes that are tailored to the youth's consumption habits has created a policy vacuum, which leaves a crucial demographic at risk of tobacco addiction and its consequent health hazards, undermining broader public health efforts within the region to curtail the tobacco epidemic.

When considering the implications of the current study's findings and in determining suitable steps forward, it is crucial to acknowledge that the socioeconomic and political landscapes of ASEAN countries are incredibly diverse. In some countries, smoking is not only a social habit but also an integral part of cultural heritage.^69^, ^70^ Children and adolescents often observe authority figures smoking, reinforcing its social acceptance. Offering cigarettes is considered a gesture of respect and a sign of courteous exchange.^71^ Addressing the challenges of the tobacco epidemic requires tailored country-specific approaches. Nevertheless, some universal strategies still apply when adapting interventions to individual country needs. Increasing taxation on tobacco products is an underutilised intervention in almost all ASEAN countries, despite it being recognised as one of the most effective tactics for reducing tobacco use. Currently, there are notable gaps in the pricing and taxation of tobacco products across the region.^72^ However, in countries with robust tobacco taxation, notable progress has been observed. For instance, in the Philippines, efforts to enforce taxation policies have led to substantial improvements, with a substantial decline in smoking prevalence among both males and females over the past 30 years. In contrast, in countries with weaker taxation policies, such as Laos and Indonesia, smoking prevalence has either increased or remained unchanged. As numerous studies have highlighted, increases in tobacco taxation not only generate short-term and medium-term revenue gains, but the long-term public health improvements will also result in substantial societal and fiscal benefits.^73^, ^74^ In addition, expanding public smoking bans and offering national cessation programmes are two other areas in which many ASEAN nations have not yet reached the highest standard and can devote further effort to protect their populations from tobacco exposure and assist current smokers in quitting. Aside from country-specific strategies, it is imperative for the ASEAN community to stand united against the influence of the tobacco industry. Collective effort is required to combat misinformation and restrict tobacco companies’ sponsorship and CSR activities. Regional strategy is needed to monitor and eliminate illicit trade to prevent the industry from manipulating market supply and evading taxation. This unified front is crucial for fostering a healthier ASEAN and sustaining the region's socioeconomic growth.^12^, ^43^, ^75^

There are a few limitations to the current study. First, information on smoking status was collected through individual or household surveys, which are subject to self-report bias, with respondents potentially misreporting their true smoking behaviour. Second, the availability of data across the study period varied by country and age group. In particular, smoking behaviours among younger age groups are not routinely monitored in every country and are often limited to youth in school settings. Sparse data necessitated reliance on modelling for estimates, which introduced higher levels of uncertainty. To address these data gaps, robust methods were used to exploit spatial and temporal correlations in tobacco use trends, as well as proven sociodemographic covariates to derive reliable predictions. However, the resulting estimates need to be interpreted with caution. These data challenges highlight the need to strengthen surveillance, particularly in countries where the burden of smoking remains high. Third, the definition of smoking varied between surveys, and although established methods were applied to correct and adjust for this discrepancy,^9^ there might still be unaccounted biases. Fourth, survey sampling methodology is not always representative of the nation. For instance, surveys for youth tobacco smoking typically target youth in school settings, thereby excluding those who are outside the school system or have dropped out. Furthermore, some national surveys—such as those in Singapore—excluded a large proportion of migrant workers, a group known for higher smoking prevalence (31% current smokers^76^), causing skewed national statistics. Fifth, although pack-years is a widely used metric for assessing cumulative smoking exposure, it does not distinguish between the effects of smoking intensity and duration, which might have differing impacts on specific outcomes (eg, COPD).^77^, ^78^ Finally, the current study focused solely on smoking tobacco and excluded other forms of tobacco consumption, such as chewing tobacco. This limitation is especially relevant in countries such as Cambodia and Myanmar (chewing tobacco is highly prevalent among Cambodian women and Myanmarese men).^79^ Moreover, the impact of ENDS is not captured in the current analysis due to an absence of available data. The use of ENDS has only begun to be monitored routinely in the past decade.^80^, ^81^ Our understanding of the relative risks associated with ENDS on various health outcomes is still limited.^82^ As more robust data become available, future studies on smoking burden could incorporate this evidence. The ASEAN countries have pledged commitment to the UN Sustainable Development Goals, with Goal 3.4 specifically targeting a one-third reduction in premature mortality associated with NCDs through prevention and treatment. Inextricably linked to reaching Goal 3.4 is Goal 3.A, which advocates for strengthening the implementation and enforcement of the WHO Framework Convention on Tobacco Control across all countries. This study reveals a mixed pattern of progress across the ASEAN. If the member countries are to achieve the 2030 Sustainable Development Goal targets, they must amplify their efforts to counteract tobacco use.

### GBD 2021 ASEAN Tobacco Collaborators

### Affiliations

### Contributors

### Data sharing

To download the input data used in these analyses, please visit the Global Health Data Exchange GBD 2021 website (https://ghdx.healthdata.org/gbd-2021/sources). All results from this study are publicly accessible. To download estimates produced in these analyses, please visit the GBD Results tool (https://vizhub.healthdata.org/gbd-results/).

## Declaration of interests

JHVT reports a leadership or fiduciary role in other board, society, committee or advocacy group, paid or unpaid as the co-founder of Benang Merah Research Center, outside the submitted work. All other authors report no competing interests.
