# Supplementary material for: The epidemiology and burden of smoking in countries of the Association of Southeast Asian Nations (ASEAN), 1990–2021: findings from the Global Burden of Disease Study 2021
Source: Lancet Public Health. 2025 May 27;10(6):e442–55. doi: 10.1016/S2468-2667(24)00326-8 (PMC12127265; doi:10.1016/S2468-2667(24)00326-8)
Supplement: Supplementary appendix [file mmc1.pdf]

THE LANCET
Public Health

Supplementary appendix

This appendix formed part of the original submission and has been peer reviewed. We post it as supplied by the authors.

Supplement to: GBD 2021 ASEAN Tobacco Collaborators. The epidemiology and burden of smoking in countries of the Association of Southeast Asian Nations (ASEAN), 1990–2021: findings from the Global Burden of Disease Study 2021. *Lancet Public Health* 2025; **10:** e442–55.

Supplementary appendix to “The epidemiology and burden of smoking in countries of the Association of Southeast Asian Nations: findings from the Global Burden of Disease Study 2021”

This appendix provides further methodological detail and supplementary results for “The Epidemiology and Burden of Smoking in the ASEAN Countries: Findings from the Global Burden of Disease Study 2021.”

Preamble

This appendix includes further methodological detail for "The Epidemiology and Burden of Smoking in the ASEAN Countries: Findings from the Global Burden of Disease Study 2021." This study complies with the Guidelines for Accurate and Transparent Reporting (GATHER) recommendations.[1] It includes detailed tables and information on data in an effort to maximise transparency in our estimation process and provide a comprehensive description of analytical steps. We intend this appendix to be a living document, to be updated with each iteration of the Global Burden of Disease Study.

Portions of this appendix have been reproduced or adapted from appendices for Ng et al.,[2] Reitma et al.,[3] Zheng et al.,[4,5] GBD 2021 Causes of Death Collaborators,[6] GBD 2021 Disease and Injuries Collaborators [7] and GBD 2021 Risk Factors Collaborators.[8] References are provided for reproduced or adapted sections.

Table of Contents

Supplementary appendix to "The Epidemiology and Burden of Smoking in the ASEAN Countries: Findings from the Global Burden of Disease Study 2021" 1
- Preamble 2
- Data Sources Used for Smoking Prevalence Estimation in GBD 2021 4
- Model flow chart 25
- Exposure 25
  - Case Definition 25
  - Data Inclusion Criteria 25
  - Data Extraction 26
  - Adjustment for Non-Standard Case Definition 26
  - Age and Sex Splitting 27
  - Smoking Prevalence Modeling 27
  - Estimation of Dose-Response Exposure Among Current and Former Smokers 28
  - Dose-Response risk curves for 36 risk-Outcome Pairs using Burden of Proof Approach 28
  - PAF Calculation 30
  - Attributable burden calculation 30
- Supplementary Results 32
- GATHER Checklist 49
- PRISMA Flowcharts 51
- Author Contributions 52
- References 54

Data Sources Used for Smoking Prevalence Estimation in GBD 2021

Figure SM1 shows the distribution of data across geographical locations. For the 10 ASEAN countries, a total of 159 data sources were used. Figure SM2 illustrates the volume of available data year and countries; Table SM1 lists the sources of data from ASEAN countries; and Figure SM3 shows the systematic review process for studies and data sources specific to ASEAN countries.

Figure SM1 Number of data sources used to estimate current smoking prevalence 1980-2021

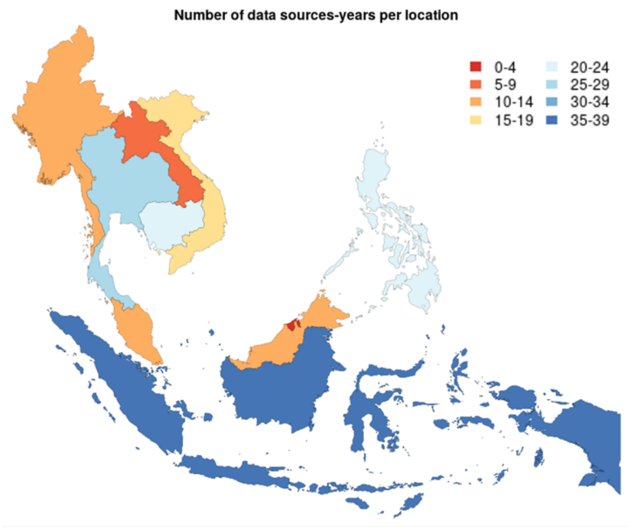

Figure SM2 Number of data sources used to estimate current smoking prevalence included from the 10 ASEAN Countries 1980-2021

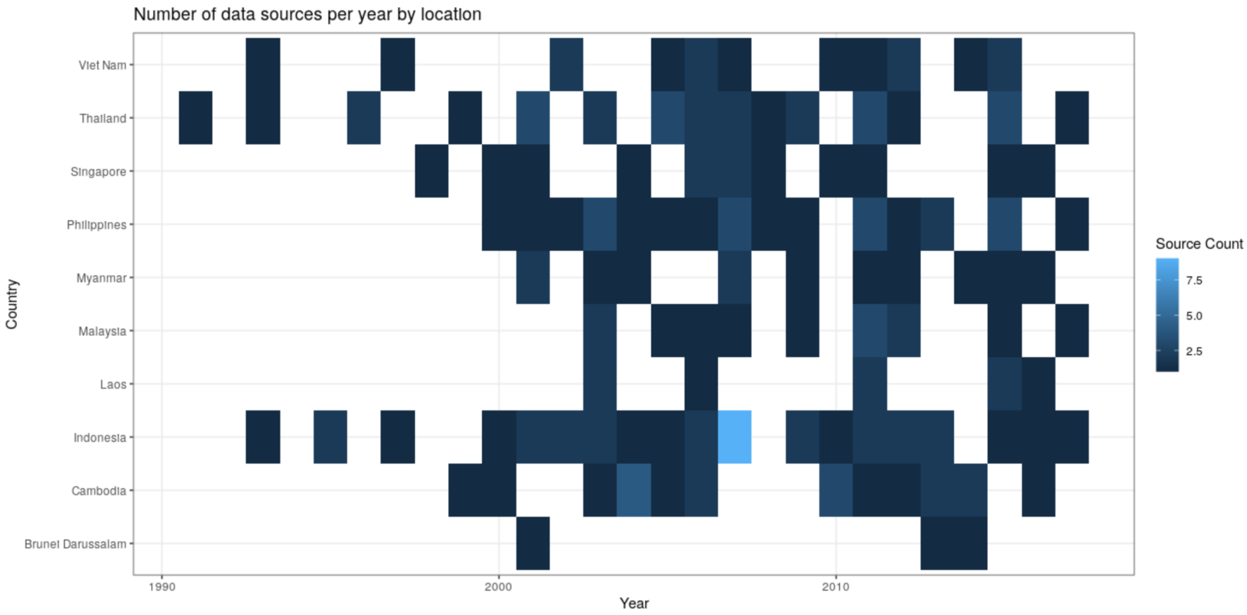

Table SM1 List of data sources included to estimate current smoking prevalence from the 10 ASEAN countries

NID	Locations	Years	Source Citation
1598	Brunei Darussalam	2001	Department of Economic Planning and Development (Brunei Darussalam). Brunei Population and Housing Census 2001.
5583	Indonesia	1993	RAND Corporation, University of Indonesia. Indonesia Family Life Survey 1993-1994. Santa Monica, United States of America: RAND Corporation.
5827	Indonesia	1997	Macro International, Inc, RAND Corporation, University of California, Los Angeles (UCLA), University of Indonesia. Indonesia Family Life Survey 1997.
6111	Indonesia	2000	Center for Population and Policy Studies, Gadjah Mada University (Indonesia), RAND Corporation. Indonesia Family Life Survey 2000. Santa Monica, United States of America: RAND Corporation.
6464	Indonesia	2007	Center for Population and Policy Studies, Gadjah Mada University (Indonesia), RAND Corporation, SurveyMETER. Indonesia Family Life Survey 2007-2008. Santa Monica, United States of America: RAND Corporation.
6473	Indonesia	2006	Centers for Disease Control and Prevention (CDC) and World Health Organization (WHO). Indonesia Global Youth Tobacco Survey 2006. Atlanta, United States: Centers for Disease Control and Prevention (CDC).
6687	Indonesia	1995	Central Bureau of Statistics (Indonesia), Ministry of Health (Indonesia), United Nations Children's Fund (UNICEF). Indonesia National Socioeconomic Survey 1995.
6842	Indonesia	2001	Central Bureau of Statistics (Indonesia), Ministry of Health (Indonesia), World Bank. Indonesia National Socioeconomic Survey 2001.
6874	Indonesia	2003	Statistics Indonesia. Indonesia National Socioeconomic Survey 2003.

NID	Locations	Years	Source Citation
6904	Indonesia	2004	Statistics Indonesia. Indonesia National Socioeconomic Survey 2004.
6970	Indonesia	2007	Statistics Indonesia. Indonesia National Socioeconomic Survey 2007.
8919	Myanmar	2004	Centers for Disease Control and Prevention (CDC) and World Health Organization (WHO). Myanmar Global Youth Tobacco Survey 2004. Atlanta, United States: Centers for Disease Control and Prevention (CDC).
10924	Philippines	2004	Centers for Disease Control and Prevention (CDC) and World Health Organization (WHO). Philippines Global Youth Tobacco Survey 2004. Atlanta, United States: Centers for Disease Control and Prevention (CDC).
12705	Thailand	2005	Centers for Disease Control and Prevention (CDC), World Health Organization (WHO). Thailand Global Youth Tobacco Survey 2005. Atlanta, United States of America: Centers for Disease Control and Prevention (CDC).
12765	Thailand	2003	Ministry of Public Health (Thailand). Thailand National Health and Examination Survey 2003-2004.
13550	Viet Nam	2007	Centers for Disease Control and Prevention (CDC) and World Health Organization (WHO). Viet Nam Global Youth Tobacco Survey 2007. Atlanta, United States: Centers for Disease Control and Prevention (CDC).
13572	Viet Nam	1993	World Bank (WB), General Statistics Office (Viet Nam). Viet Nam Living Standards Measurement Survey 1992-1993. Washington D.C., United States: World Bank (WB)
13614	Viet Nam	2002	General Statistics Office (Viet Nam), United Nations Development Programme (UNDP), World Bank (WB). Viet Nam Living Standards Measurement Survey 2002. General Statistical Office, World Bank.

NID	Locations	Years	Source Citation
19156	Cambodia	2000	Macro International, Inc, Ministry of Health (Cambodia), National Institute of Statistics (Cambodia). Cambodia Demographic and Health Survey 2000. Fairfax, United States of America: ICF International.
19167	Cambodia	2005	Macro International, Inc, National Institute of Public Health (Cambodia), National Institute of Statistics (Cambodia). Cambodia Demographic and Health Survey 2005-2006. Fairfax, United States of America: ICF International.
20011	Indonesia	2002	Macro International, Inc, Ministry of Health (Indonesia), National Family Planning Coordinating Board (Indonesia), Statistics Indonesia. Indonesia Demographic and Health Survey 2002-2003. Fairfax, United States of America: ICF International.
20021	Indonesia	2007	Macro International, Inc, Ministry of Health (Indonesia), National Family Planning Coordinating Board (Indonesia), Statistics Indonesia. Indonesia Demographic and Health Survey 2007. Fairfax, United States of America: ICF International.
20040	Indonesia	2002, 2003	Macro International, Inc, Statistics Indonesia. Indonesia Special Demographic and Health Survey 2002-2003. Fairfax, United States of America: ICF International.
20699	Philippines	2003	Macro International, Inc, National Statistics Office (Philippines). Philippines Demographic and Health Survey 2003. Fairfax, United States of America: ICF International.
21421	Philippines	2008	Macro International, Inc, National Statistics Office (Philippines). Philippines Demographic and Health Survey 2008. Fairfax, United States of America: ICF International, 2010.
21692	Lao People's Democratic Republic	2003	World Health Organization (WHO). Laos World Health Survey 2003.

NID	Locations	Years	Source Citation
21721	Malaysia	2003	World Health Organization (WHO). Malaysia World Health Survey 2003. Geneva, Switzerland: World Health Organization (WHO), 2005.
21769	Myanmar	2003	World Health Organization (WHO). Myanmar World Health Survey 2003. Geneva, Switzerland: World Health Organization (WHO), 2005.
21819	Philippines	2003	World Health Organization (WHO). Philippines World Health Survey 2003. Geneva, Switzerland: World Health Organization (WHO), 2005.
21938	Viet Nam	2002	World Health Organization (WHO). Vietnam World Health Survey 2002-2003. Geneva, Switzerland: World Health Organization (WHO), 2005.
21998	Philippines	2009	CDC Foundation, Centers for Disease Control and Prevention (CDC), Department of Health (Philippines), Johns Hopkins Bloomberg School of Public Health, National Statistics Office (Philippines), Research Triangle Institute, Inc. (RTI), World Health Organization (WHO). Philippines Global Adult Tobacco Survey 2009. Atlanta, United States of America: Centers for Disease Control and Prevention (CDC).
22008	Thailand	2009	Baqai Institute of Diabetology and Endocrinology (BIDE) (Pakistan), Centers for Disease Control and Prevention (CDC), Faculty of Public Health at Mahidol University (Thailand), Health Systems Research Institute (Thailand), Ministry of Public Health (Thailand), National Statistical Office (Thailand), Tobacco Control Research and Knowledge Management Center (Thailand), World Health Organization (WHO). Thailand Global Adult Tobacco Survey 2009. Atlanta, United States of America: Centers for Disease Control and Prevention (CDC), 2011.

NID	Locations	Years	Source Citation
22022	Viet Nam	2010	Bloomberg Philanthropies, CDC Foundation, Centers for Disease Control and Prevention (CDC), General Statistics Office (Vietnam), Hanoi Medical University, Ministry of Health (Vietnam), World Health Organization (WHO). Vietnam Global Adult Tobacco Survey 2010.
22680	Indonesia	2007	Agency of Health Research and Development (Indonesia). Indonesia Basic Health Research 2007-2008.
25940	Viet Nam	2006	General Statistics Office (Vietnam), United Nations Development Programme (UNDP), World Bank. Vietnam Living Standards Measurement Survey 2006.
26869	Indonesia	2007	Macro International, Inc, Statistics Indonesia. Indonesia Special Demographic and Health Survey 2007. Fairfax, United States of America: ICF International.
28312	Cambodia	2003	Centers for Disease Control and Prevention (CDC) and World Health Organization (WHO). Cambodia Global Youth Tobacco Survey 2003. United States: Centers for Disease Control and Prevention (CDC), 2003.
28849	Malaysia	2003	Centers for Disease Control and Prevention (CDC) and World Health Organization (WHO). Malaysia Global Youth Tobacco Survey 2003. United States: Centers for Disease Control and Prevention (CDC), 2003.
29067	Myanmar	2001	Centers for Disease Control and Prevention (CDC) and World Health Organization (WHO). Myanmar Global Youth Tobacco Survey 2001. United States: Centers for Disease Control and Prevention (CDC), 2001.
29072	Myanmar	2007	Centers for Disease Control and Prevention (CDC) and World Health Organization (WHO). Myanmar Global Youth Tobacco Survey 2007. United States: Centers for Disease Control and Prevention (CDC), 2007.

NID	Locations	Years	Source Citation
29276	Philippines	2000	Centers for Disease Control and Prevention (CDC) and World Health Organization (WHO). Philippines Global Youth Tobacco Survey 2000. United States: Centers for Disease Control and Prevention (CDC), 2000.
29281	Philippines	2007	Centers for Disease Control and Prevention (CDC) and World Health Organization (WHO). Philippines Global Youth Tobacco Survey 2007. United States: Centers for Disease Control and Prevention (CDC), 2007.
29371	Singapore	2000	Centers for Disease Control and Prevention (CDC) and World Health Organization (WHO). Singapore Global Youth Tobacco Survey 2000. United States: Centers for Disease Control and Prevention (CDC), 2000.
30057	Viet Nam	2005	World Health Organization (WHO). Vietnam - H·ªì Ch√≠ Minh STEPS Noncommunicable Disease Risk Factors Survey 2005.
30107	Malaysia	2005	Ministry of Health (Malaysia), World Health Organization (WHO). Malaysia STEPS Noncommunicable Disease Risk Factors Survey 2005-2006.
30160	Indonesia	2001	Ministry of Health (Indonesia), World Health Organization (WHO). Indonesia STEPS Noncommunicable Disease Risk Factors Survey 2001.
30235	Indonesia	2010	Statistics Indonesia. Indonesia National Socioeconomic Survey 2010.
30379	Cambodia	2010	ICF Macro, Ministry of Health (Cambodia), National Institute of Statistics (Cambodia). Cambodia Demographic and Health Survey 2010-2011. Fairfax, United States of America: ICF International.
30842	Cambodia	1999	National Institute of Statistics (Cambodia), United Nations Development Programme (UNDP), World Bank. Cambodia Socio-Economic Survey 1999. Phnom Penh,

NID	Locations	Years	Source Citation
			Cambodia: National Institute of Statistics (Cambodia).
30963	Cambodia	2004	National Institute of Statistics (Cambodia), Statistics Sweden. Cambodia Socio-Economic Survey 2003-2005. Phnom Penh, Cambodia: National Institute of Statistics (Cambodia).
31050	Cambodia	2006	National Institute of Statistics (Cambodia), Statistics Sweden. Cambodia Socio-Economic Survey 2006-2007. Phnom Penh, Cambodia: National Institute of Statistics (Cambodia).
43552	Indonesia	2009	Statistics Indonesia. Indonesia National Socioeconomic Survey 2009.
46781	Viet Nam	1997	General Statistics Office (Vietnam), World Bank. Vietnam Living Standards Measurement Survey 1997-1998. Washington DC, United States of America: World Bank.
58421	Singapore	2004	Ministry of Health (Singapore). Singapore National Health Survey 2004.
76705	Indonesia	2012	ICF International, Ministry of Health (Indonesia), National Population and Family Planning Board (Indonesia), Statistics Indonesia. Indonesia Demographic and Health Survey 2012. Fairfax, United States of America: ICF International.
95428	Thailand	1991	Ministry of Public Health (Thailand). Thailand National Health Examination Survey 1991-1992.
103977	Philippines	2002	Carolina Population Center, University of North Carolina at Chapel Hill, Office of Population Studies, University of San Carlos (Philippines). Philippines - Cebu Longitudinal Health and Nutrition Survey 2002-2003. Chapel Hill, United States of America: Carolina Population Center, University of North Carolina at Chapel Hill.

NID	Locations	Years	Source Citation
104027	Philippines	2005	Carolina Population Center, University of North Carolina at Chapel Hill, Office of Population Studies, University of San Carlos (Philippines). Philippines - Cebu Longitudinal Health and Nutrition Survey 2004-2006. Chapel Hill, United States of America: Carolina Population Center, University of North Carolina at Chapel Hill.
104843	Indonesia	2011	Agency of Health Research and Development (Indonesia), Centers for Disease Control and Prevention (CDC), Ministry of Health (Indonesia), Statistics Indonesia, World Health Organization (WHO). Indonesia Global Adult Tobacco Survey 2011. Atlanta, United States of America: Centers for Disease Control and Prevention (CDC), 2014.
104844	Malaysia	2011	Centers for Disease Control and Prevention (CDC), Department of Statistics (Malaysia), Institute for Public Health, Ministry of Health (Malaysia), International Islamic University Malaysia, Johns Hopkins Bloomberg School of Public Health, Ministry of Health (Malaysia), Research Triangle Institute, Inc. (RTI), University of Malaya, World Health Organization (WHO). Malaysia Global Adult Tobacco Survey 2011.
107333	Cambodia	2010	Ministry of Health (Cambodia), University of Health Sciences (Cambodia), World Health Organization (WHO). Cambodia STEPS Noncommunicable Disease Risk Factors Survey 2010.
107334	Myanmar	2009	Ministry of Health (Myanmar), World Health Organization Regional Office for South-East Asia (SEARO). Myanmar STEPS Noncommunicable Disease Risk Factors Survey 2009.
108748	Indonesia	2007	Centers for Disease Control and Prevention (CDC), Ministry of Education (Indonesia), Ministry of Health (Indonesia), World Health

NID	Locations	Years	Source Citation
			Organization (WHO). Indonesia Global School-Based Student Health Survey 2007.
108815	Myanmar	2007	Centers for Disease Control and Prevention (CDC), Joint United Nations Program on HIV/AIDS (UNAIDS), United Nations Children's Fund (UNICEF), United Nations Educational, Scientific and Cultural Organization (UNESCO), World Health Organization (WHO). Myanmar Global School-Based Student Health Survey 2007. Geneva, Switzerland: World Health Organization (WHO).
108818	Philippines	2003	Centers for Disease Control and Prevention (CDC), World Health Organization (WHO). Philippines Global School-Based Student Health Survey 2003 . Geneva, Switzerland: World Health Organization (WHO).
108819	Philippines	2007	Centers for Disease Control and Prevention (CDC), World Health Organization (WHO). Philippines Global School-Based Student Health Survey 2007. Geneva, Switzerland: World Health Organization (WHO).
109960	Cambodia	2010	Centers for Disease Control and Prevention (CDC), World Health Organization (WHO). Cambodia Global Youth Tobacco Survey 2010. Atlanta, United States of America: Centers for Disease Control and Prevention (CDC).
110365	Indonesia	2009	Centers for Disease Control and Prevention (CDC), World Health Organization (WHO). Indonesia Global Youth Tobacco Survey 2009. Atlanta, United States of America: Centers for Disease Control and Prevention (CDC), 2013.
110370	Lao People's Democratic Republic	2011	Centers for Disease Control and Prevention (CDC), World Health Organization (WHO). Laos Global Youth Tobacco Survey 2011. Atlanta, United States of America: Centers for Disease Control and Prevention (CDC), 2013.

NID	Locations	Years	Source Citation
110381	Myanmar	2011	Centers for Disease Control and Prevention (CDC), World Health Organization (WHO). Myanmar Global Youth Tobacco Survey 2011. Atlanta, United States of America: Centers for Disease Control and Prevention (CDC).
110384	Thailand	2009	Centers for Disease Control and Prevention (CDC), World Health Organization (WHO). Thailand Global Youth Tobacco Survey 2009. Atlanta, United States of America: Centers for Disease Control and Prevention (CDC).
110387	Malaysia	2009	Centers for Disease Control and Prevention (CDC), World Health Organization (WHO). Malaysia Global Youth Tobacco Survey 2009. Atlanta, United States of America: Centers for Disease Control and Prevention (CDC).
110392	Philippines	2011	Centers for Disease Control and Prevention (CDC), World Health Organization (WHO). Philippines Global Youth Tobacco Survey 2011. Atlanta, United States of America: Centers for Disease Control and Prevention (CDC), 2013.
111887	Cambodia	2004	National Institute of Statistics (Cambodia). Cambodia Smoking Behavior Survey 2004.
112140	Thailand	2001	National Statistical Office (Thailand). Thailand Cigarette Smoking and Drinking Behavior Survey 2001.
112142	Thailand	2007	National Statistical Office (Thailand). Thailand Cigarette Smoking and Drinking Behavior Survey 2007.
112184	Singapore	2001	Ministry of Health (Singapore). Singapore National Health Surveillance Survey 2001.
112185	Singapore	1998	Ministry of Health (Singapore). Singapore National Health Survey 1998.
112186	Singapore	2007	Ministry of Health (Singapore). Singapore National Health Surveillance Survey 2007.
112214	Thailand	1996, 1999, 2001	Ministry of Health (New Zealand), Statistics New Zealand. New Zealand Health Survey

NID	Locations	Years	Source Citation
			1996-1997. Wellington, New Zealand: Statistics New Zealand.
120199	Thailand	2008	Centers for Disease Control and Prevention (CDC), Joint United Nations Program on HIV/AIDS (UNAIDS), Ministry of Education (Thailand), Ministry of Public Health (Thailand), United Nations Children's Fund (UNICEF), United Nations Educational, Scientific and Cultural Organization (UNESCO), World Health Organization (WHO). Thailand Global School-Based Student Health Survey 2008. Geneva, Switzerland: World Health Organization (WHO).
124581	Indonesia	2005	National Narcotics Board (Indonesia), University of Indonesia. Indonesia National Survey on Drug Abuse and Illicit Drugs 2005.
126178	Myanmar	2001	Ministry of Health (Myanmar), World Bank, World Health Organization (WHO). Tobacco Economics in Myanmar. Washington DC, United States of America: World Bank, 2003.
126199	Thailand	2011	National Statistical Office (Thailand). Thailand Cigarette Smoking and Drinking Behavior Survey 2011. Bankok, Thailand: National Statistical Office (Thailand).
126397	Singapore	2010	Ministry of Health (Singapore). Singapore National Health Survey 2010.
130015	Malaysia	2012	Centers for Disease Control and Prevention (CDC), Ministry of Health (Malaysia), World Health Organization (WHO). Malaysia Global School-Based Student Health Survey 2012.
130017	Philippines	2011	Centers for Disease Control and Prevention (CDC), World Health Organization (WHO). Philippines Global School-Based Student Health Survey 2011.

NID	Locations	Years	Source Citation
130022	Viet Nam	2012	Centers for Disease Control and Prevention (CDC), World Health Organization (WHO). Vietnam Global School-Based Student Health Survey 2012-2013. Geneva, Switzerland: World Health Organization (WHO).
135505	Cambodia	2004	Institute for Social Research, University of Michigan. Cambodia Elderly Survey 2004. Ann Arbor, United States of America: Institute for Social Research, University of Michigan.
136037	Cambodia, Indonesia, Philippines, Thailand	2004, 1995, 2007, 2001, 1996, 2003	World Health Organization (WHO). WHO Global Infobase - Tobacco Use Prevalence. Geneva, Switzerland: World Health Organization (WHO).
142107	Singapore	2006	Health Promotion Board (Singapore). Singapore Student Health Survey 2006.
142943	Philippines	2013	ICF International, Philippines Statistics Authority. Philippines Demographic and Health Survey 2013. Fairfax, United States of America: ICF International, 2014.
150912	Thailand	1993	National Statistical Office (Thailand). Thailand Survey of Cigarette Smoking Behavior 1993.
151015	Thailand	2005	Ministry of Public Health (Thailand). Thailand Noncommunicable Disease and Injury Behavior Risk Surveillance Survey 2005.
151016	Thailand	2007	Ministry of Public Health (Thailand). Thailand Noncommunicable Disease and Injury Behavior Risk Surveillance Survey 2007.
154215	Indonesia	2007	National Institute of Health Research and Development (NIHRD), Ministry of Health (Indonesia). Analysis of National and Subnational (Regional, Province) Socioeconomic Determinants of Tobacco Use and Tobacco Related Diseases in Indonesia.
155317	Brunei Darussalam	2014	Centers for Disease Control and Prevention (CDC), Ministry of Health (Brunei Darussalam), World Health Organization (WHO). Brunei

NID	Locations	Years	Source Citation
			Global School-Based Student Health Survey 2014.
155318	Cambodia	2013	Centers for Disease Control and Prevention (CDC), Ministry of Health (Cambodia), World Health Organization (WHO). Cambodia Global School-Based Student Health Survey 2013. Atlanta, United States of America: Centers for Disease Control and Prevention (CDC).
157024	Cambodia	2014	ICF International, Ministry of Health (Cambodia), National Institute of Statistics (Cambodia). Cambodia Demographic and Health Survey 2014. Fairfax, United States of America: ICF International, 2017.
157061	Myanmar	2015	ICF International, Ministry of Health and Sports (Myanmar). Myanmar Demographic and Health Survey 2015-2016. Fairfax, United States of America: ICF International, 2017.
220942	Indonesia	2013	Agency of Health Research and Development (Indonesia), KNCV Tuberculosis Foundation, Ministry of Health (Indonesia), SRL Adelaide, United States Agency for International Development (USAID), World Health Organization (WHO). Indonesia Tuberculosis Prevalence Survey 2013-2014.
230382	Thailand	2005	Institute for Population and Social Research, Mahidol University (Thailand), International Tobacco Control Policy Evaluation Project, Thai Health Promotion Foundation, University of Waterloo (Canada). Thailand International Tobacco Control Survey 2005.
230383	Thailand	2006	Institute for Population and Social Research, Mahidol University (Thailand), International Tobacco Control Policy Evaluation Project, Thai Health Promotion Foundation, University of Waterloo (Canada). Thailand International Tobacco Control Survey 2006.

NID	Locations	Years	Source Citation
231764	Cambodia	2013	Centers for Disease Control and Prevention (CDC), Ministry of Social Affairs, Veterans and Youth Rehabilitation (MoSVY) (Cambodia), Ministry of Women's Affairs (Cambodia), National Institute of Statistics (Cambodia), Together for Girls, United Nations Children's Fund (UNICEF). Cambodia Violence Against Children Survey 2013. Washington, DC, United States of America: Together for Girls.
237392	Thailand	2011	Action on Smoking and Health Foundation (Thailand), Centers for Disease Control and Prevention (CDC), Faculty of Public Health at Mahidol University (Thailand), Health Systems Research Institute (Thailand), Ministry of Public Health (Thailand), National Statistical Office (Thailand), Tobacco Control Research and Knowledge Management Center (Thailand), World Health Organization (WHO). Thailand Global Adult Tobacco Survey 2011.
237970	Indonesia	2015	Centers for Disease Control and Prevention (CDC), World Health Organization (WHO). Indonesia Global School-Based Student Health Survey 2015. Geneva, Switzerland: World Health Organization (WHO).
237975	Thailand	2015	Centers for Disease Control and Prevention (CDC), World Health Organization (WHO). Thailand Global School-Based Student Health Survey 2015. Geneva, Switzerland: World Health Organization (WHO).
238317	Lao People's Democratic Republic	2015	Centers for Disease Control and Prevention (CDC), World Health Organization (WHO). Laos Global School-Based Student Health Survey 2015.
249262	Philippines	2013	Food and Nutrition Research Institute, Department of Science and Technology (Philippines). Philippines National Nutrition Survey 2013-2014.

NID	Locations	Years	Source Citation
250035	Myanmar	2014	Ministry of Health (Myanmar), Myanmar Medical Association, World Health Organization (WHO). Myanmar STEPS Noncommunicable Disease Risk Factors Survey 2014. Geneva, Switzerland: World Health Organization (WHO).
256541	Malaysia	2015	Institute for Public Health, Ministry of Health (Malaysia). Malaysia National Health and Morbidity Survey 2015.
256543	Malaysia	2011	Institute for Public Health, Ministry of Health (Malaysia). Malaysia National Health and Morbidity Survey 2011.
265136	Thailand	2015	Ministry of Public Health (Thailand). Thailand Noncommunicable Disease and Injury Behavior Risk Surveillance Survey 2015.
265163	Indonesia	2013	CDC Foundation, Centers for Disease Control and Prevention (CDC), National Institute of Health Research and Development (NIHRD) (Indonesia), World Health Organization (WHO). Indonesia Global Youth Tobacco Survey 2013-2014. Atlanta, United States of America: Centers for Disease Control and Prevention (CDC).
282087	Indonesia	2016	Central Bureau of Statistics (Indonesia). Indonesia National Socioeconomic Survey 2016.
287143	Philippines	2015	CDC Foundation, Centers for Disease Control and Prevention (CDC), Department of Health (Philippines), Johns Hopkins Bloomberg School of Public Health, Philippine Statistics Authority, Research Triangle Institute, Inc. (RTI), World Health Organization (WHO). Philippines Global Adult Tobacco Survey 2015. Atlanta, United States of America: Centers for Disease Control and Prevention (CDC).

NID	Locations	Years	Source Citation
288487	Viet Nam	2014	Centers for Disease Control and Prevention (CDC), Vietnam Steering Committee on Smoking and Health (VINACOSH), World Health Organization (WHO). Vietnam Global Youth Tobacco Survey 2014. Atlanta, United States of America: Centers for Disease Control and Prevention (CDC).
289822	Thailand	2015	Centers for Disease Control and Prevention (CDC), Department of Disease Control, Ministry of Public Health (Thailand), World Health Organization (WHO). Thailand Global Youth Tobacco Survey 2015. Atlanta, United States of America: Centers for Disease Control and Prevention (CDC).
289967	Philippines	2015	Centers for Disease Control and Prevention (CDC), Epidemiology Bureau, Department of Health (Philippines), World Health Organization (WHO). Philippines Global Youth Tobacco Survey 2015. Atlanta, United States of America: Centers for Disease Control and Prevention (CDC).
293973	Viet Nam	2015	Ministry of Health (Vietnam). Vietnam STEPS Noncommunicable Disease Risk Factors Survey 2015. Geneva, Switzerland: World Health Organization (WHO), 2016.
294139	Viet Nam	2015	CDC Foundation, Centers for Disease Control and Prevention (CDC), General Statistics Office (Vietnam), Hanoi Medical University, Ministry of Health (Vietnam), RTI International, Vietnam Steering Committee on Smoking and Health (VINACOSH), World Health Organization (WHO). Vietnam Global Adult Tobacco Survey 2015.
315363	Philippines	2015	Centers for Disease Control and Prevention (CDC), World Health Organization (WHO). Philippines Global School-Based Student Health Survey 2015. Geneva, Switzerland: World Health Organization (WHO), 2020.

NID	Locations	Years	Source Citation
337877	Philippines	2017	ICF International, Philippines Statistics Authority, United States Agency for International Development (USAID). Philippines Demographic and Health Survey 2017. Fairfax, United States of America: ICF International, 2018.
344549	Cambodia	2006	Gallup. Cambodia World Poll 2005-2006.
344579	Indonesia	2006	Gallup. Indonesia World Poll 2005-2006.
344592	Lao People's Democratic Republic	2006	Gallup. Laos World Poll 2005-2006.
344599	Malaysia	2006	Gallup. Malaysia World Poll 2005-2006.
344616	Philippines	2006	Gallup. Philippines World Poll 2005-2006.
344627	Singapore	2006	Gallup. Singapore World Poll 2005-2006.
344639	Thailand	2006	Gallup. Thailand World Poll 2005-2006.
344650	Viet Nam	2006	Gallup. Vietnam World Poll 2005-2006.
344699	Indonesia	2007	Gallup. Indonesia World Poll 2007.
344714	Malaysia	2007	Gallup. Malaysia World Poll 2007.
344732	Philippines	2007	Gallup. Philippines World Poll 2007.
344739	Singapore	2007	Gallup. Singapore World Poll 2007.
344866	Singapore	2008	Gallup. Singapore World Poll 2008.
345443	Cambodia	2011	Gallup. Cambodia World Poll 2011.
345479	Indonesia	2011	Gallup. Indonesia World Poll 2011.
345493	Lao People's Democratic Republic	2011	Gallup. Laos World Poll 2011.
345503	Malaysia	2011	Gallup. Malaysia World Poll 2011.
345526	Philippines	2011	Gallup. Philippines World Poll 2011.
345537	Singapore	2011	Gallup. Singapore World Poll 2011.
345552	Thailand	2011	Gallup. Thailand World Poll 2011.

NID	Locations	Years	Source Citation
345566	Viet Nam	2011	Gallup. Vietnam World Poll 2011.
345840	Cambodia	2012	Gallup. Cambodia World Poll 2012.
345876	Indonesia	2012	Gallup. Indonesia World Poll 2012.
345898	Malaysia	2012	Gallup. Malaysia World Poll 2012.
345907	Myanmar	2012	Gallup. Myanmar World Poll 2012.
345920	Philippines	2012	Gallup. Philippines World Poll 2012.
345946	Thailand	2012	Gallup. Thailand World Poll 2012.
345958	Viet Nam	2012	Gallup. Vietnam World Poll 2012.
347926	Brunei Darussalam	2013	Centers for Disease Control and Prevention (CDC), Ministry of Health (Brunei Darussalam), World Health Organization (WHO). Brunei Darussalam Global Youth Tobacco Survey 2013. Atlanta, United States of America: Centers for Disease Control and Prevention (CDC).
347931	Cambodia	2016	Centers for Disease Control and Prevention (CDC), Ministry of Health (Cambodia), World Health Organization (WHO). Cambodia Global Youth Tobacco Survey 2016. Atlanta, United States of America: Centers for Disease Control and Prevention (CDC).
347949	Lao People's Democratic Republic	2003	Centers for Disease Control and Prevention (CDC), World Health Organization (WHO). Laos Global Youth Tobacco Survey 2003.
347951	Lao People's Democratic Republic	2016	Centers for Disease Control and Prevention (CDC), Ministry of Health (Laos), Southeast Asia Tobacco Control Alliance (SEATCA), World Health Organization (WHO). Laos Global Youth Tobacco Survey 2016. Atlanta, United States of America: Centers for Disease Control and Prevention (CDC), 2016.
349280	Myanmar	2016	Centers for Disease Control and Prevention (CDC), Ministry of Health and Sports (Myanmar), World Health Organization (WHO). Myanmar Global Youth Tobacco Survey 2016.

NID	Locations	Years	Source Citation
			Atlanta, United States of America: Centers for Disease Control and Prevention (CDC).
350797	Malaysia	2017	Family Health Development Division, Ministry of Health (Malaysia), Institute for Public Health, Ministry of Health (Malaysia), Ministry of Education (Malaysia), Ministry of Health (Malaysia). Malaysia National Health and Morbidity Survey 2017.
395694	Indonesia	2017	Central Bureau of Statistics (Indonesia). Indonesia National Socioeconomic Survey 2017. Jakarta, Indonesia: Central Bureau of Statistics (Indonesia), 2018.
420659	Cambodia, Lao People's Democratic Republic, Thailand, Singapore	2014, 2015, 2017, 2016	World Health Organization (WHO). WHO Report on the Global Tobacco Epidemic 2019. Geneva, Switzerland: World Health Organization (WHO), 2019.

Figure SM3 GBD 2021 systematic review flow chart for studies and data sources specific to ASEAN countries

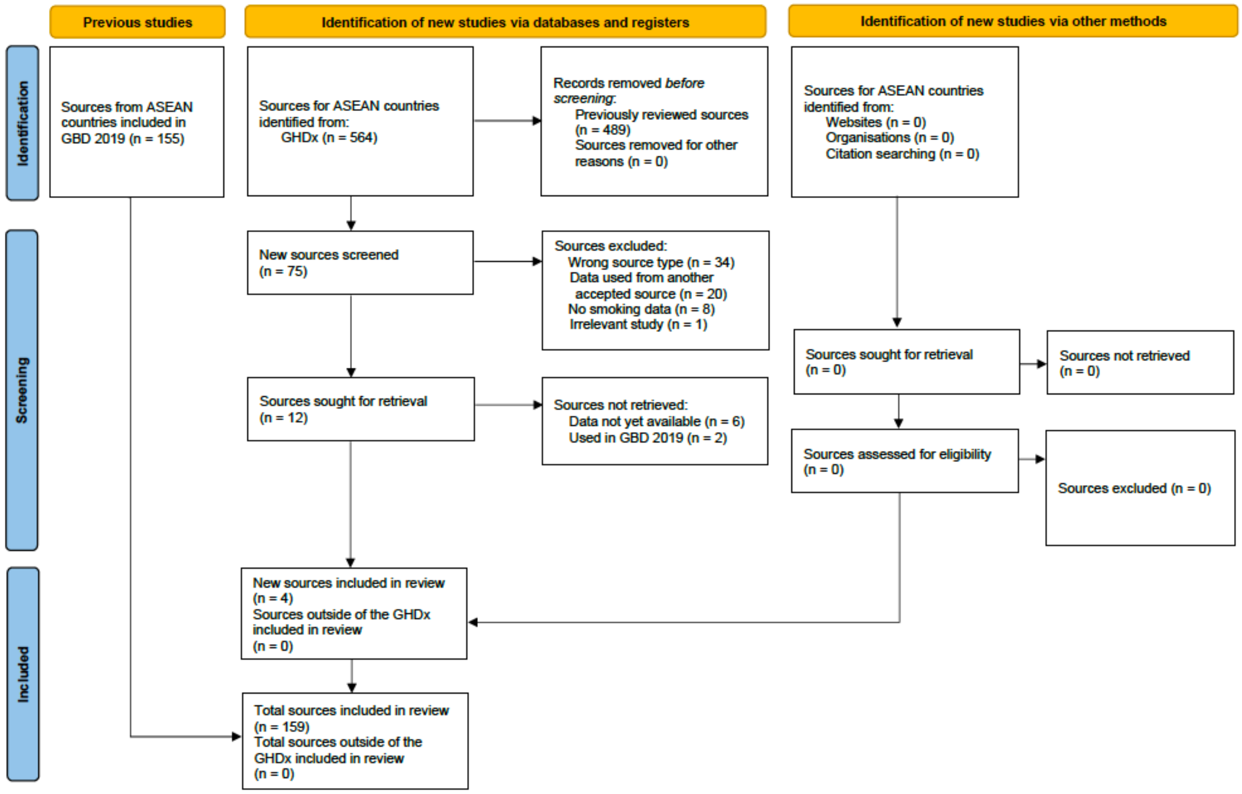

Model flow chart

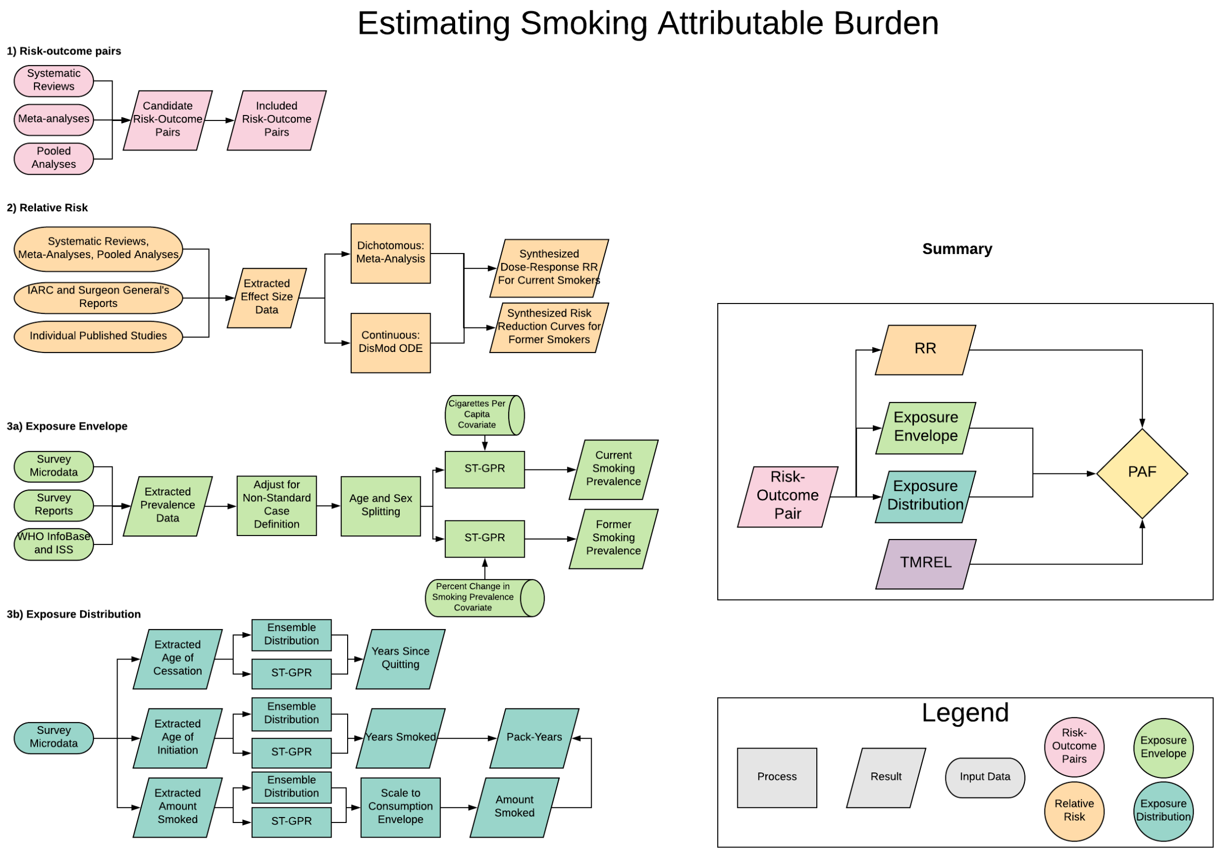

Exposure

Case Definition

Current smokers are defined as individuals who, at the time of survey, use any smoked tobacco product on a daily or occasional basis. This includes products such as factory-made and hand-rolled cigarettes, cigars, pipes, hookahs, bidis, and any other types of smoked tobacco. On the other hand, former smokers are defined as individuals who have abstained from all forms of smoked tobacco for a minimum of 6 months or based on the specific definitions used by a survey. Depending on survey data availability, an additional criterion of a minimum of 100 equivalents of cigarettes lifetime consumption is applied to the definition.

Data Inclusion Criteria

We performed a comprehensive review of surveys that provided information on tobacco usage. All data included in this study were accessible on the Global Health Data Exchange (GHDx, http://ghdx.healthdata.org/). Surveys were considered for inclusion if they met the following criteria:

- Inclusion of measures of tobacco use that either conform with to our case definitions, or could be adjusted to fit these definitions using previously established crosswalk strategies.[3]
- Surveys where data gathering took place between January 1, 1980, and December 31, 2021.
- Representative samples of the general population of any one of the 204 nations and territories included in our study

- Surveys exclusively conducted amongst specific sub-groups such as those with a particular disease, racial/ethnic minorities, pregnant women, etc. were excluded with the exception of school-based survey. Most studies focusing on youth tobacco use rely on data collected from school settings. Given the scarcity of youth smoking data, to ensure broad data coverage for youth aged 10-17, we chose to include school-based surveys.
- Only self-report tobacco use data are included. Data reported by a proxy respondent was excluded.
- Data must be for individuals aged 10 years and above.

Data Extraction

We applied data extraction strategies consistent with prior GBD burden of tobacco use studies.[3] Specifically, using primary data from both individual-level microdata and survey report summaries, we extract details on current, former, or ever-smoked tobacco usage. All combinations of usage frequency (daily, occasional, and unspecified) and types of smoked tobacco products used (all smoked tobacco, cigarettes, hookah, and other smoked products such as cigars or pipes) were considered. Other variants of tobacco products, such as hand-rolled cigarettes, were classified into the aforementioned four categories based on product similarities. This resulted in 36 possible combinations (3 tobacco use frequency x 4 tobacco product types x 3 estimation time periods). The present study only considered smoked tobacco products. Smoked drugs and smokeless tobacco were evaluated separately as distinct risk factors. Novel nicotine delivery systems, such as e-cigarettes and heated tobacco products, were not included in GBD 2021 due to insufficient historical data on exposure and health impacts.

We retrieved demographic data from the microdata, including details such as age, sex, location, and yea along with survey metadata like survey weights, primary sampling units, and strata. This enabled us to aggregate individual-level data into the standard GBD five-year age-sex groups and derive reliable uncertainty estimates. In instances where survey design indicators were not specified in the microdata, we adopted a conservative design effect of 2·25. As for survey reports, we extracted data at the most granular age-sex group available.

We included a total of 2806 nationally representative cross-sectional household surveys to assess the prevalence of both current and former smoking. With regards to the 10 ASEAN countries, 159data sources were utilised in total. The number of data sources applied for each country is depicted in Figure SM1. Among the 204 countries and territories analysed, 200 (98%) had at least one data source, and 171 (84%) had at least five data sources. The most recent data source for 44% of the countries was either from 2017 or 2018. The data density and sources from the 10 ASEAN countries are presented in Figure SM2 and Table SM1.

A comprehensive list of data sources can be accessed via the GBD 2021 Data Input Sources Tool: http://ghdx.healthdata.org/gbd-2019/data-input-sources.

Adjustment for Non-Standard Case Definition

Adjustments were made to data points which deviated from the standard GBD smoking case definition of current and former usage of any smoked tobacco product. Leveraging data sources which provided information based on multiple case definitions, adjustment coefficients were derived and applied to

convert the required data points. Details of the methodologies and values of coefficients can be found in previous publication.[3]

Age and Sex Splitting

For survey report data that supplied information for age groups broader than the GBD standard 5-year categories, or data that was reported by aggregating both sexes, we employed the enhanced modelling approach established in GBD 2019[3] to disaggregate data by 5-year age groups, separately for males and females. Specifically, a spatiotemporal Gaussian process regression (ST-GPR) model was developed using a training dataset to approximate age-sex patterns. Subsequently, the data that were reported with combined sex and in broad age groups were disaggregated based on the approximated age-sex pattern. The model was designed with the flexibility to allow for variations in age-sex patterns by country and year. The prediction errors that stemmed from the age and sex splitting process was carried forward to subsequent prevalence modelling stages, leading to a more accurate reflection of estimation uncertainties.

Smoking Prevalence Modeling

To model current and former smoking prevalence, we employed spatiotemporal Gaussian process regression (ST-GPR). A detailed account of the ST-GPR method is provided in previous publications. In short, the mean function of the GPR was derived through a two-step process: an estimation of a linear model of mean prevalence and a smoothing function of the residuals.

For the estimation of current smoking prevalence, the linear function, which is estimated by a mixed-effects model, is defined as:

$$logit(p_{l,a,t}) = \beta_0 + \beta_1 CPC_{l,t} + \sum_{k=2}^{19} \beta_k I_{A[a]} + \alpha_s + \alpha_r + \alpha_l + \epsilon_{l,a,t} \ ,$$

where $p_{l,a,t}$ refers to the prevalence of current smoker at location l, for age group a, at year t; $CPC_{l,t}$ is the tobacco consumption covariate at location l in year t; $I_{A[a]}$ is a dummy variable indicating the specific age group A that the prevalence point $p_{l,a,t}$ captures, and α_s, α_r, and α_l are the random intercepts for super region, region and location respectively. The model was fitted separately by sex. Following the linear modelling step, a locally weighted polynomial regression (LOESS) was applied to the resulting residuals to capture variabilities which were not adequately accounted for. The LOESS involves customized weights to reflect spatiotemporal correlation and age pattern in the data. The combination of linear model and smoothing function serves the mean function input to GPR and the covariate function of the GPR is defined using Matérn Covariance function (See details in Reistma 2021[3])

A similar strategy was used for estimating former smoking prevalence, with the deviation in the estimation of linear model which is defined as follows:

$$logit\left({p^f}_{l,a,t}\right) = \beta_0 + \beta_1(\%\Delta p_{l,a,t}) + \beta_2 p_{l,a,t} + \sum_{k=2}^{19} \beta_k I_{A[a]} + \alpha_s + \alpha_r + \alpha_l + \epsilon_{l,a,t} \ ,$$

Where ${p^f}_{l,a,s,t}$ is the prevalence of former smoker at location l, for age group a at year t; $(\%\Delta p_{l,a,s,t})$ refers to the percent change in current smoking prevalence between year t and year $t-1$; $p_{a,l,s,t}$ refers to the current smoking prevalence at location l in super region s, for age group a at year t derived from the ST-FPR model for current smoking

Estimation of Dose-Response Exposure Among Current and Former Smokers

Consistent with GBD 2019, two continuous metrics, the cigarette equivalents per smoker per day and pack-years, were used as proxies for exposure among current smokers. The cigarette equivalent per smoker per day metric reflects smoking intensity by capturing daily tobacco consumption, whereas the pack-years metric reflects both the duration and quantity of smoking. A single pack-year corresponds to smoking a pack of cigarettes (20 cigarettes per pack) daily for one year.

The mean of each metric was estimated using household survey and supply-side separately. In particular, with household surveys, simulation models were developed to approximate individual smoking history and predict consumption patterns. Subsequently, the *mean* cigarette equivalents per smoker per day and *mean* pack-years by geographical location, years, age, and sex were estimated. A limitation of household survey data is the potential underestimation of consumption due to inaccurate self-reporting. To address this issue, domestic supply-side consumption data were considered.

Four sources of supply-side data were used, namely, the United Nations Food and Agriculture Organization (1961-2013, domestic supply), the United States Department of Agriculture (1960-2005, domestic supply), and Euromonitor (2002-2016, retail supply). Several steps were involved in order to derive the mean cigarette equivalents per smoker per day and mean pack-years by geographical location, years, age and sex. The incorporation of supply-side data involved several steps. First, data ere carefully vetted to identify and remove outliers. Second, imputations were performed to fill in data gaps and derive complete time series from each data source for every country. Third, given that supply-side consumption volumes were reported at the country level, the data were redistributed proportionally according to the mean consumption pattern predicted from survey estimates previously derived.

The final mean cigarettes equivalents per smoker per day and mean pack-years metrics were calculated by averaging the respective estimates from household-survey results and supply-side approximation. A full distribution of each metrics, based on an ensemble of distribution functions, were subsequently derived using the estimated means. Details of the estimation process can be found in Reistma et al.[3]

Dose-Response risk curves for 36 risk-Outcome Pairs using Burden of Proof Approach

Based on casual evidence in the literature, 36 risk-outcome pairs were included in the estimation of smoking-related burden: tuberculosis, lower respiratory tract infections, esophageal cancer, stomach cancer, bladder cancer, liver cancer, laryngeal cancer, lung cancer, breast cancer, cervical cancer, colorectal cancer, lip and oral cancer, nasopharyngeal cancer, other pharyngeal cancer, pancreatic cancer, kidney cancer, leukemia, ischemic heart disease, stroke, atrial fibrillation and flutter, aortic aneurysm, peripheral arterial disease, chronic obstructive pulmonary disease, other chronic respiratory diseases, asthma, peptic ulcer disease, gallbladder and biliary tract diseases, Alzheimer disease and other dementias, Parkinson disease (protective), multiple sclerosis, type-II diabetes, rheumatoid arthritis, low back pain, cataracts, macular degeneration, and fracture. Updated systematic reviews were performed to obtain data on the risk of smoking in relation to each of the health outcomes.

The risk for all risk-outcome pairs, except for the risk of fractures, was assessed assuming continuous levels of smoking exposure defined by pack-year, cigarettes smoked daily per smoker, and duration since quitting. As for the risk of fractures, binary exposure to smoking comparing smoker versus non-smoker/never smoked was assumed.

Dose-response curves for each outcome were derived using the latest Burden of Proof approach – meta-regression, Bayesian, regularized, trimmed (MR-BRT).[4,5] MR-BRT is a meta-analytic approach that is used to derive risk functions. Building upon the classic mixed-effects meta-regression structure, MR-BRT incorporates a Bayesian regularized spline function to allow for more flexible modelling of non-linear risk curves. A set of covariates and random effects were included to reflect biases in study design, within-study correlation and between-study heterogeneity. To enhance the robustness of the regression estimates, the least trimmed squares was applied to mitigate the impact of outliers. An adjustment strategy based on Fisher Scoring correction was employed to account for data sparsity for specific risk-outcome pairs.

The derivation of dose-response graphs can be summarised in six steps: (1) Systematic data extraction: Relative-risk data were systematically identified and extracted from published studies using a standardised methodology; (2) Modelling exposure-risk relationship: The relationship between exposure and relative risk was estimated and integrated over the full range of exposure levels across comparison groups; (3) Bias assessment and adjustment: Systematic biases related to study characteristics were examined and adjusted to ensure more accurate estimates; (4) Quantifying heterogeneity and within-study correlation: Between- and within-study variances were assessed and adjusted; (5) Evaluation of small-study effects: The potential for publication or reporting bias was evaluated by assessing evidence for small-study effects; (6) Estimation of risk curve: A risk curve was derived to provide a conservative interpretation of the average risk increase across the supported exposure range. Details of the risk-outcome pairs and related modelling parameters and results can be found in Zheng et al. (2021).[5]

The exposure levels for the risk curves for each outcome was assumed to range from zero to 100 pack-years or cigarettes per smoker per day. Relative risks were presumed identical for both sexes for all outcomes, except for breast cancer, cervical cancer, and prostate cancer, where sex-specific assumptions were applied. Different age-specific assumptions were also applied for non-cardiovascular (non-CVD) disease outcomes versus cardiovascular disease (CVD) outcomes including stroke, ischemic heart disease, atrial fibrillation and flutter, aortic aneurysm, and peripheral arterial disease. Specifically, for non-CVD outcomes, we assumed a uniform risk curve across all ages. In contrast, considering the impact of smoking on CVD outcomes is known to plateau with age, age-specific risk curves were assumed for all CVD outcomes.

Age-specific risk curves were estimated by first deriving a reference dose-response risk of smoking for each CVD outcome using relative risk (RR) data irrespective of age. Subsequently, the corresponding age group of which the reference risk curve represented was determined by the weighted mean age of all RR data inputs. Different approaches were used in the calculation of mean age for case-control versus cohort studies. For cohort studies, this calculation included both baseline mean age and mean/median years of follow-up. For case-control studies, we utilized the reported mean age at baseline.

Age pattern of excess risk of smoking on CVD outcomes were modelled separately using a streamed-lined MR-BRT to model the age pattern of excess risk (i.e., RR-1) of smoking on CVD outcomes. The model utilized age-group specific RR data and relevant bias covariates from the literature. A spline was used to capture non-linear age pattern, random effects were used to capture variability between studies, and bias covariates were included to capture systematic differences in exposure types (i.e. current, former and ever smoker).

By comparing the estimated age pattern of excess risk with the reference risk curve for each CVD outcome, age attenuation factors (AF) of excess risk were derived. Finally, by applying the AF to the reference curve, we obtain the age-group specific risk curves for each CVD outcomes. Details of the calculation can be found in Dai et al. [9]

PAF Calculation

Population attributable fractions were calculated using a formula that considers the prevalence of never, former, and current smokers, the distribution of years since quitting among former smokers, and the relative risk for years since quitting and for cigarette-equivalents per smoker per day or pack-years. The specific calculation is as follows:

$$PAF = \frac{p^n + p^f \int \exp(x) rr(x) + p^c \int \exp(y) rr(y) - 1}{p^n + p^f \int \exp(x) rr(x) + p^c \int \exp(y) rr(y)},$$

where p^n, p^f, p^c refers to the prevalence of never smokers, former smokers, and current smokers respectively; $\exp(x)$ is the distribution of years since quitting among former smokers; $rr(x)$ is the relatively risk with respect to years since quitting. $\exp(y)$ is the distribution of exposure defined either by cigarettes-equivalent per smoker per day or pack-years, and $rr(y)$is the relative risk for cigarette-equivalents per smoker per day or pack-years.

The exposure metrics used differ based on health outcomes. For cancers and chronic respiratory diseases, both the duration and dose of exposure are considered to have significant impact on risk, pack-years was used as the exposure metric. As for cardiovascular diseases and other health outcomes, with which dose is of higher relevance compared to duration, cigarette-equivalents per smoker per day was used.

Distinct from GBD 2019, in order to reduce algorithmic complexity, exposure was no longer adjusted to account for time lag in the effect of smoking on health outcomes. Comparison of results from current study with prior findings indicated considerable consistency, implying that the stream-lined approach remained robust.

Attributable burden calculation

The total smoking-attributable burden, measured in terms of DALYs, was calculated as follows:

$$DALY_{aslt}^{Smoking} = \sum_{o=1}^{36} DALY_{aslto} \times PAF_{aslto},$$

where $DALY_{aslt}^{Smoking}$ refers to the total smoking attributable DALYs for a given age group a, sex s, location l and year t was derived from the summation of outcome-specific DALYs multiplied by the corresponding PAF. Similar formula was used to derive the total smoking attributable YLL, YLD and mortality as described below:

$$YLL_{aslt}^{Smoking} = \sum_{o=1}^{36} YLL_{aslto} \times PAF_{aslto},$$

$$YLD_{aslt}^{Smoking} = \sum_{o=1}^{36} YLD_{aslto} \times PAF_{aslto},$$

$$Deaths_{aslt}^{Smoking} = \sum_{o=1}^{36} Deaths_{aslto} \times PAF_{aslto},$$

All the burden metrics, $DALY_{aslto}$, YLL_{aslto}, YLD_{aslto} and $DALY_{aslto}$ were calculated using methods detailed in previous publications.[6,7]

Supplementary Results

Figure SM4 Annualised rate of change in (A) number of tobacco smokers and (B) age-standardised prevalence of smoking tobacco use. The solid line represents the ASEAN average, the grey lines represent the 10 ASEAN countries.

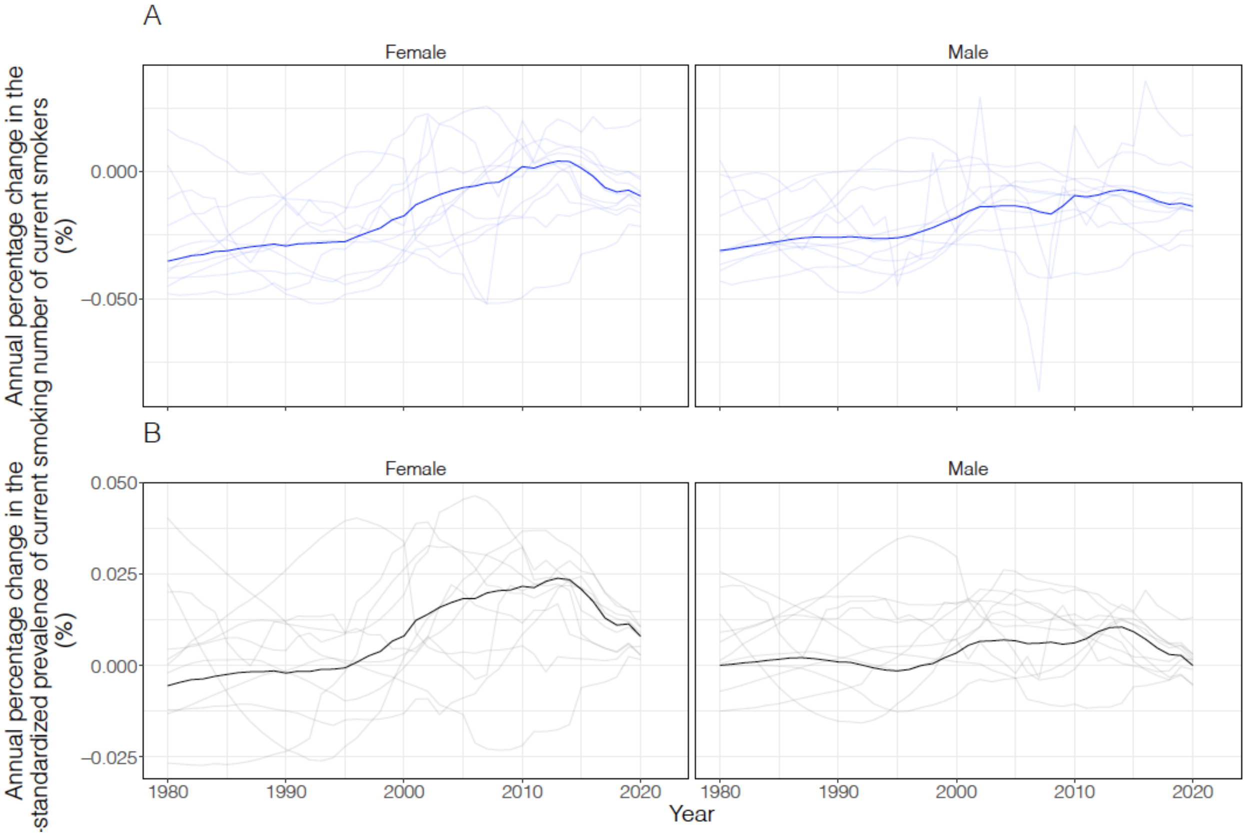

Figure SM5 Proportion of all-cause deaths that were attributable to smoking tobacco use among (A) females and (B) males of all ages in 2021

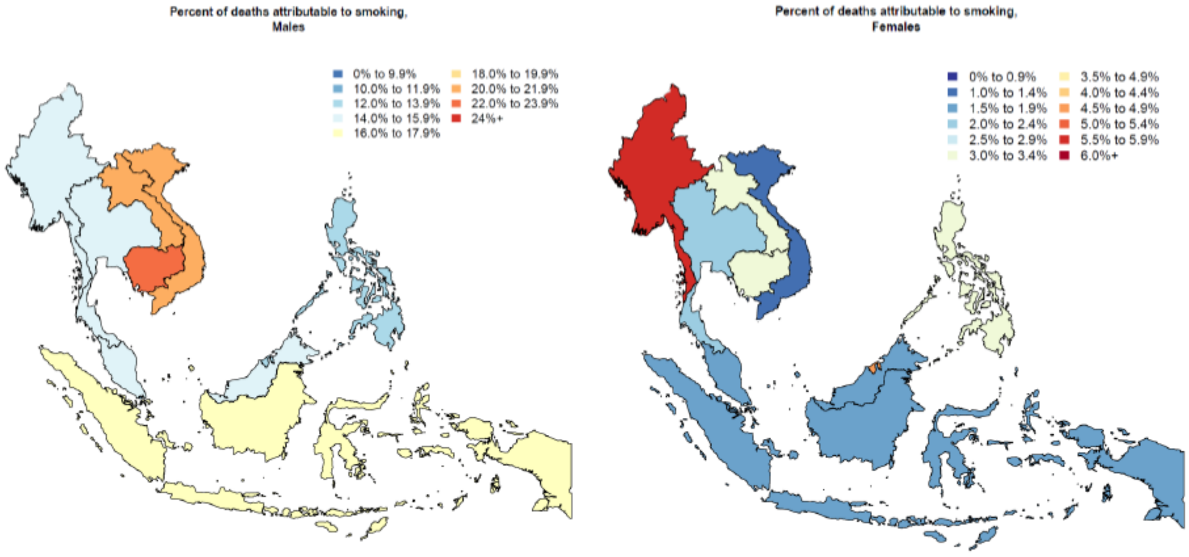

Table SM2 Number of current smokers aged 15+ (millions) in 1990, 2021 and percent change between 1990 – 2021, by sex, for ASEAN countries

	Both sexes			Females			Males		
	Number (millions)		% Change in number	Number (millions)		% Change in number	Number(millions)		% Change in number
	1990	2021	1990 - 2021	1990	2021	1990 - 2021	1990	2021	1990 – 2021
ASEAN Region	83·9 (82·2, 85·5)	137 (134, 139)	63·3 (59·0, 67·8)	8·12 (7·41, 8·83)	11·6 (10·6, 12·8)	42·8 (25·7, 62·9)	75·8 (74·2, 77·2)	125 (123, 127)	65·5 (61·3, 70·0)
Brunei Darussalam	0·05 (0·04, 0·05)	0·06 (0·06, 0·07)	30·4 (16·2, 45·6)	0·01 (0·00, 0·01)	0·01 (0·01, 0·01)	45·7 (3·2, 106)	0·04 (0·04, 0·04)	0·05 (0·05, 0·06)	28·6 (13·4, 44·7)
Cambodia	1·20 (1·13, 1·26)	2·51 (2·38, 2·66)	110 (93·0, 128)	0·15 (0·12, 0·18)	0·33 (0·27, 0·41)	127 (63·2, 204)	1·05 (0·99, 1·11)	2·18 (2·06, 2·31)	108 (90·9, 126)
Indonesia	33·7 (32·6, 34·9)	66·9 (65·0, 68·9)	98·5 (89·8, 108)	1·80 (1·46, 2·20)	3·72 (3·02, 4·63)	110 (52·5, 181)	31·9 (30·8, 33·0)	63·2 (61·4, 64·9)	98·0 (89·7, 106)
Lao People's Democratic Republic	0·53 (0·49, 0·57)	1·37 (1·29, 1·45)	160 (137, 184)	0·06 (0·05, 0·08)	0·16 (0·13, 0·20)	183 (105, 293)	0·47 (0·44, 0·50)	1·21 (1·14, 1·27)	158 (134, 183)
Malaysia	3·09 (2·90, 3·29)	5·51 (5·16, 5·86)	78·8 (62·7, 95·9)	0·23 (0·17, 0·29)	0·35 (0·28, 0·44)	58·4 (7·7, 124)	2·86 (2·67, 3·05)	5·16 (4·82, 5·50)	80·6 (64·1, 98·9)
Myanmar	8·76 (8·27, 9·28)	9·37 (8·90, 9·89)	7·1 (-1·2, 15·7)	1·69 (1·41, 1·98)	1·71 (1·41, 2·03)	2·2 (-19·9, 28·1)	7·08 (6·70, 7·47)	7·66 (7·25, 8·09)	8·4 (0·0, 16·9)
Philippines	13·0 (12·2, 13·7)	19·3 (18·3, 20·3)	48·7 (38·5, 60·5)	2·18 (1·76, 2·65)	3·02 (2·56, 3·53)	39·7 (7·3, 78·8)	10·8 (10·2, 11·4)	16·2 (15·4, 17·1)	50·8 (40·4, 62·1)
Singapore	0·48 (0·44, 0·53)	0·67 (0·61, 0·73)	39·6 (21·5, 57·3)	0·12 (0·10, 0·16)	0·15 (0·12, 0·19)	22·3 (-12·6, 70·5)	0·36 (0·32, 0·39)	0·52 (0·47, 0·57)	46·2 (27·6, 65·9)
Thailand	11·3 (10·7, 11·9)	12·0 (11·4, 12·5)	6·0 (-1·6, 13·7)	1·16 (0·96, 1·40)	1·08 (0·88, 1·33)	-5·8 (-29·5, 24·5)	10·1 (9·60, 10·7)	10·9 (10·3, 11·4)	7·4 (-0·4, 15·5)
Viet Nam	11·8 (11·3, 12·3)	19·3 (18·6, 20·1)	63·6 (54·8, 74·0)	0·73 (0·59, 0·87)	1·04 (0·81, 1·29)	43·4 (3·2, 90·2)	11·1 (10·6, 11·5)	18·3 (17·6, 19·1)	65·0 (55·7, 75·3)

Table SM3 Total Cigarette-equivalents and cigarette-equivalents per smoker aged 15 years and older in 1990 and 2021, for the 10 ASEAN countries

Location	Total Cigarette-equivalents (billion units)		Cigarette-equivalents per smoker aged 15+	
	1990	2021	1990	2021
ASEAN region	354 (340, 368)	566 (539, 596)	4220 (4080, 4360)	4140 (3960, 4340)
Brunei Darussalam	0·35 (0·29, 0·42)	0·36 (0·28, 0·45)	7340 (6250, 8700)	5700 (4700, 7040)
Cambodia	6·06 (5·58, 6·54)	14·6 (12·0, 18·2)	5060 (4760, 5380)	5810 (4800, 7190)
Indonesia	134 (124, 145)	280 (257, 304)	3980 (3680, 4290)	4190 (3860, 4530)
Lao People's Democratic Republic	2·43 (2·24, 2·64)	5·19 (4·52, 5·98)	4600 (4380, 4860)	3790 (3360, 4320)
Malaysia	15·0 (13·7, 16·4)	23·0 (20·9, 25·1)	4870 (4570, 5190)	4170 (3910, 4440)
Myanmar	31·1 (27·4, 35·4)	24·0 (20·2, 28·8)	3550 (3180, 3970)	2560 (2170, 3050)
Philippines	63·3 (58·5, 68·6)	81·4 (73·7, 89·9)	4880 (4570, 5190)	4230 (3900, 4620)
Singapore	2·65 (2·25, 3·10)	3·21 (2·86, 3·56)	5520 (4830, 6320)	4790 (4480, 5100)
Thailand	46·2 (42·9, 49·8)	45·9 (42·1, 50·2)	4100 (3860, 4340)	3840 (3580, 4160)
Viet Nam	52·6 (48·2, 57·7)	88·8 (80·5, 98·2)	4450 (4100, 4830)	4590 (4190, 5030)

Table SM4 All-cause and all-age smoking attributable deaths among people aged 30+ by location and sex, 2021· Estimates are reported as total number of attributable deaths, percent of deaths attributable to smoking, and smoking attributable death rate (per 100 000), for the 10 ASEAN countries

All-cause and all-age smoking attributable deaths by location and sex, 2021

Location	Sex	Total number of deaths attributable to smoking (95% uncertainty interval)	Percent of deaths attributable to smoking (95% uncertainty interval)	Smoking-attributable death rate per 100 000 (95% uncertainty interval)
ASEAN region	Female	49200 (37700, 62300)	2·32 (1·78, 2·98)	27·9 (21·4, 35·4)
	Male	477000 (394000, 562000)	17·4 (14·4, 20·7)	281 (232, 331)
Brunei Darussalam	Female	37·5 (26·3, 51·1)	4·89 (3·54, 6·63)	33·0 (23·2, 45·0)
	Male	148 (116, 186)	15·7 (12·6, 18·8)	118 (92·6, 148)
Cambodia	Female	1630 (1050, 2340)	3·02 (2·05, 4·26)	41·2 (26·4, 59·2)
	Male	12800 (9660, 15900)	22·8 (18·0, 27·7)	370 (279, 460)
Indonesia	Female	15500 (9990, 21700)	1·79 (1·25, 2·53)	21·8 (14·0, 30·5)
	Male	208000 (156000, 265000)	18·6 (14·3, 22·7)	293 (221, 373)
Lao People's Democratic Republic	Female	558 (367, 785)	3·17 (2·27, 4·39)	36·7 (24·1, 51·6)
	Male	4720 (3490, 5980)	20·9 (16·4, 25·0)	313 (231, 396)
Malaysia	Female	1220 (819, 1720)	1·40 (0·93, 2·01)	16·0 (10·8, 22·7)
	Male	18000 (14900, 21200)	14·3 (11·9, 16·9)	221 (183, 260)
Myanmar	Female	10700 (7350, 15300)	5·34 (3·73, 7·61)	75·9 (51·9, 108)
	Male	36300 (27700, 45700)	15·3 (11·9, 19·4)	299 (228, 377)

Philippines	Female	10700 (7640, 14300)	3·26 (2·50, 4·20)	43·9 (31·5, 58·9)
	Male	62300 (46800, 79700)	13·7 (11·2, 16·0)	259 (194, 331)
Singapore	Female	2728 (176, 424)	2·59 (1·66, 3·96)	14·1 (8·98, 21·6)
	Male	1440 (1160, 1730)	11·4 (9·26, 13·6)	69·4 (56·1, 83·4)
Thailand	Female	4890 (3190, 7250)	1·84 (1·27, 2·61)	20·8 (13·6, 30·8)
	Male	52000 (37600, 70100)	15·4 (12·3, 18·7)	247 (178, 333)
Viet Nam	Female	3660 (2430, 5340)	1·27 (0·88, 1·79)	13·1 (8·67, 19·1)
	Male	80900 (62200, 97500)	21·6 (18·0, 25·3)	309 (238, 373)

Table SM5 All-cause and all-age smoking attributable disability-adjusted life-years (DALYs) among people aged 30+ by location and sex, 2021· Estimates are reported as total number of attributable DALYs, percent of DALYs attributable to smoking, and smoking attributable DALY rate (per 100 000), for the 10 ASEAN countries

All-cause and all-age smoking attributable DALYs by location and sex, 2021				
Location	Sex	Total number of DALYs attributable to smoking, thousands (95% uncertainty interval)	Percent of DALYs attributable to smoking (95% uncertainty interval)	Smoking-attributable DALY rate per 100 000 (95% uncertainty interval)
ASEAN region	Female	1340 (1020, 1720)	1·76 (1·33, 2·27)	760 (577, 977)
	Male	14300 (11800, 16900)	15·1 (12·5, 17·8)	8440 (6950, 9960)
Brunei Darussalam	Female	1·20 (0·88, 1·68)	3·45 (2·38, 4·61)	1073 (718, 1474)
	Male	5·30(4·18, 6·72)	12·4 (9·78, 15·3)	4210 (3320, 5340)
Cambodia	Female	47·0 (31·1, 67·5)	2·45 (1·69, 3·48)	1186 (797, 1710)
	Male	368 (278, 464)	18·3 (14·6, 22·0)	10700 (8040, 13400)
Indonesia	Female	456 (311, 641)	1·41 (0·99, 1·94)	641 (437, 902)
	Male	6440 (4860, 8270)	16·5 (13·1, 19·8)	9080 (6850, 11700)
Lao People's Democratic Republic	Female	17·2 (11·4, 23·5)	2·59 (1·84, 3·51)	1130 (750, 1540)
	Male	141 (105, 178)	17·3 (13·7, 20·7)	9310 (6970, 11800)
Malaysia	Female	29·6 (20·7, 41·6)	0·94 (0·65, 1·30)	390 (273, 548)
	Male	531 (442, 621)	12·5 (10·3, 14·7)	6520 (5420, 7620)
Myanmar	Female	265 (187, 374)	3·87 (2·78, 5·23)	1870 (1320, 2640)
	Male	976 (738, 1240)	12·4 (9·80, 15·5)	8040 (6070, 10200)
Philippines	Female	297 (215, 403)	2·55 (1·92, 3·29)	1220 (884, 1660)

	Male	2050 (1560, 2610)	12·9 (10·5, 15·2)	8530 (6480, 10800)
Singapore	Female	8·22 (5·64, 11·5)	1·80 (1·21, 2·46)	419 (287, 584)
	Male	45·0 (35·5, 55·4)	8·80 (6·89, 10·8)	2170 (1710, 2670)
Thailand	Female	120 (80·2, 171)	1·31 (0·91, 1·79)	512 (341, 728)
	Male	1410 (1030, 1900)	12·3 (9·77, 14·9)	6690 (4900, 8990)
Viet Nam	Female	98·3 (66·0, 141)	1·01 (0·71, 1·39)	350 (236, 502)
	Male	2360 (1800 2880)	18·4 (15·1, 21·3)	9010 (6870, 11000)

__Table SM__6 Smoking attributable years of life lost (YLLs), years lived with disability (YLDs), and ratios, by location for 2021, for the 10 ASEAN countries

Smoking attributable years of life lost (YLLs), years lived with disability (YLDs), and ratios, by location for 2021			
Location	YLLs (95% uncertainty interval) (thousands)	YLDs (95% uncertainty interval) (thousands)	YLL to YLD Ratio (95% uncertainty interval)
**Both sexes**			
ASEAN region	14000 (11500, 16500)	1680 (1160, 2260)	8·32 (6·48, 11·2)
Brunei Darussalam	5·24 (4·08, 6·58)	1·28 (0·83, 1·82)	4·08 (2·98, 5·67)
Cambodia	373 (275, 476)	42·0 (29·0, 58·8)	8·90 (6·27, 12·3)
Indonesia	6190 (4700, 7990)	709 (487, 964)	8·73 (6·21, 12·5)
Lao People's Democratic Republic	143 (104, 182)	15·1 (10·3, 20·8)	9·44 (6·88, 13·8)
Malaysia	499 (420, 578)	61·9 (42·7, 85·2)	8·06 (6·24, 10·8)
Myanmar	1120 (836, 1450)	121 (83·1, 166)	9·24 (7·02, 13·0)
Philippines	2100 (1620, 2650)	251 (173, 342)	8·37 (5·94, 11·8)
Singapore	39·1 (32·0, 46·6)	14·2 (9·03, 20·6)	2·76 (2·07, 3·88)
Thailand	1320 (947, 1820)	207 (143, 282)	6·37 (4·47, 9·24)
Viet Nam	2200 (1670, 2710)	258 (177, 350)	8·52 (6·34, 12·1)
**Males**			
ASEAN region	12900 (10600, 15200)	1450 (1010, 1930)	8·89 (6·95, 11·9)
Brunei Darussalam	4·30 (3·42, 5·40)	1·00 (0·65, 1·40)	4·30 (3·14, 6·08)
Cambodia	334 (249, 426)	34·6 (24·5, 47·2)	9·64 (6·85, 13·4)
Indonesia	5810 (4370, 7590)	634 (438, 850)	9·16 (6·49, 13·2)
Lao People's Democratic Republic	128 (94·2, 163)	12·6 (8·71, 17·0)	10·2 (7·38, 14·8)
Malaysia	475 (401, 550)	56·4 (39·2, 77·8)	8·42 (6·58, 11·3)
Myanmar	891 (671, 1140)	85·9 (60·7, 117)	10·4 (7·86, 14·7)
Philippines	1860 (1400, 2390)	194 (135, 260)	9·55 (6·73, 13·6)
Singapore	34·0 (27·8, 40·5)	11·0 (7·15, 15·9)	3·09 (2·37, 4·28)
Thailand	1230 (886, 1680)	182 (126, 243)	6·75 (4·77, 9·84)
Viet Nam	2120 (1610, 2600)	237 (165, 321)	8·95 (6·66, 12·8)

**Females**			
ASEAN region	1110 (852, 1410)	232 (151, 342)	4·77 (3·64, 6·56)
Brunei Darussalam	0·94 (0·66, 1·26)	0·28 (0·16, 0·43)	3·30 (2·38, 4·76)
Cambodia	39·7 (26·0, 58·0)	7·35 (4·50, 11·5)	5·39 (3·70, 7·52)
Indonesia	381 (253, 540)	75·0 (44·7, 117)	5·08 (3·53, 7·64)
Lao People's Democratic Republic	14·6 (9·60, 20·0)	2·55 (1·54, 3·97)	5·74 (4·27, 8·31)
Malaysia	24·1 (17·2, 33·2)	5·52 (3·38, 8·35)	4·36 (3·27, 5·87)
Myanmar	230 (161, 322)	35·4 (23·1, 52·6)	6·50 (4·86, 9·21)
Philippines	241 (175, 321)	56·2 (36·2, 82·3)	4·28 (3·09, 5·9)
Singapore	5·08 (3·61, 6·98)	3·14 (1·80, 4·90)	1·62 (1·12, 2·39)
Thailand	94·5 (63·0, 135)	25·7 (15·9, 38·7)	3·68 (2·56, 5·38)
Viet Nam	77·3 (52·2, 110)	21·0 (12·5, 32·3)	3·67 (2·62, 5·31)

Table SM7 Number, share, and percent change in number and share of all-age all-cause deaths attributable to smoking tobacco use, 1990-2021, for the 10 ASEAN countries

Number, share, and percent change in number and share of all-age all-cause deaths attributable to smoking tobacco use, 1990-2021, by location

Location	Number of Smoking Attributable Deaths, 2021 (thousands)	Percent Change in Number of Smoking Attributable Deaths, 1990-2021	Absolute Change in Number of Smoking Attributable deaths, 1990-2021 (thousands)	Share of Deaths Attributable to Smoking, 2021	Percent Change in Share of Deaths Attributable to Smoking, 1990-2021
ASEAN region	526 (433, 622)	78·7 (55·4, 104)	231 (167, 303)	10·8 (8·86, 12·9)	-25·6 (-35·6, -16·3)
Brunei Darussalam	0·19 (0·14, 0·23)	26·1 (3·09, 50·8)	0·04 (0·01, 0·07)	10·8 (8·58, 13·1)	-37·8 (-45·3, -29·6)
Cambodia	14·4 (10·7, 17·9)	86·1 (45·9, 134)	6·67 (3·56, 9·85)	13·1 (10·3, 15·8)	-12·8 (-26·1, 0·56)
Indonesia	223 (170, 284)	142 (84·0, 206)	131 (79·9, 185)	11·2 (8·46, 14·0)	0·85 (-21·4, 25·8)
Lao People's Democratic Republic	5·28 (3·88, 6·67)	29·4 (-1·60, 68·7)	1·16 (-0·08, 2·51)	13·1 (10·5, 15·8)	-14·1 (-28·0, -0·30)
Malaysia	19·3 (15·9, 22·8)	97·5 (80·3, 118)	9·50 (7·58, 11·6)	9·04 (7·49, 10·8)	-37·5 (-44·4, -29·5)
Myanmar	47·1 (35·4, 60·2)	-18·0 (-39·0, 8·46)	-10·9 (-26·5, 4·40)	10·7 (8·38, 13·7)	-47·3 (-56·6, -37·5)
Philippines	73·0 (56·2, 91·6)	95·8 (55·7, 141)	35·6 (20·9, 52·0)	9·32 (7·49, 11·1)	-49·0 (-55·3, -42·0)
Singapore	1·72 (1·37, 2·08)	-4·18 (-14·9, 6·50)	-0·08 (-0·27, 0·12)	7·30 (5·90, 8·96)	-47·2 (-53·2, -41·2)
Thailand	56·9 (40·9, 76·8)	52·9 (13·2, 98·7)	19·5 (5·48, 36·3)	9·44 (7·42, 11·5)	-40·1 (-50·1, -30·3)
Viet Nam	84·6 (65·3, 102)	87·1 (42·6, 137)	38·9 (21·2, 55·1)	12·8 (10·6, 15·1)	-12·7 (-22·0, 0·75)

Table SM8 Number, share, and percent change in number and share of all-age NCD deaths attributable to smoking tobacco use, 1990-2021, for the 10 ASEAN countries

Number, share, and percent change in number and share of all-age NCD-associated deaths attributable to smoking tobacco use, 1990-2021, by location

Location	Number of Smoking Attributable NCD Deaths, 2021 (thousands)	Percent Change in Number of Smoking Attributable NCD Deaths, 1990-2021	Absolute Change in Number of Smoking Attributable NCD deaths, 1990-2021 (thousands)	Share of NCD Deaths Attributable to Smoking, 2021 (percent)	Percent Change in Share of NCD Deaths Attributable to Smoking, 1990-2021
ASEAN region	463 (383, 547)	98·2 (73·1, 125)	229 (172, 294)	13·9 (11·7, 16·1)	-14·5 (-20·8, -7·80)
Brunei Darussalam	0·18 (0·14, 0·22)	31·5 (8·02, 57·7)	0·04 (0·01, 0·07)	12·4 (9·91, 14·8)	-33·1 (-41·4, -24·7)
Cambodia	11·2 (8·42, 13·8)	118 (67·5, 174)	6·05 (3·59, 8·49)	15·4 (12·8, 18·1)	-1·99 (-12·9, 11·6)
Indonesia	198 (151, 251)	185 (118, 260)	128 (83·6, 176)	14·3 (11·7, 16·9)	12·8 (-5·02, 30·0)
Lao People's Democratic Republic	4·64 (3·43, 5·85)	44·4 (8·53, 88·4)	1·39 (0·29, 2·58)	16·0 (13·5, 18·6)	-6·29 (-15·1, 2·93)
Malaysia	16·5 (13·7, 19·4)	96·7 (79·3, 120)	8·09 (6·49, 9·88)	11·9 (9·93, 13·9)	-23·2 (-29·8, -15·2)
Myanmar	42·6 (32·4, 54·1)	-6·4 (-29·9, 24·1)	-3·35 (-15·8, 9·96)	13·4 (10·8, 16·4)	-38·1 (-45·2, -30·1)
Philippines	61·0 (47·2, 76·2)	106 (63·7, 156)	31·3 (19·1, 45·5)	13·9 (11·4, 16·6)	-28·0 (-36·0, -18·7)
Singapore	1·52 (1·23, 1·83)	-6·06 (-15·8, 3·67)	-0·10 (-0·26, 0·06)	8·72 (7·07, 10·5)	-42·4 (-48·6, -36·3)
Thailand	50·3 (36·1, 68·6)	53·8 (13·2, 99·5)	17·5 (4·84, 32·7)	11·8 (9·78, 13·8)	-37·6 (-44·2, -29·8)
Viet Nam	77·2 (59·9, 92·5)	110 (60·3, 170)	40·0 (24·3, 53·8)	14·9 (12·5, 17·3)	-4·74 (-13·5, 7·48)

Table SM9 Number, share, and percent change in number and share of all-age NCD DALYs attributable to smoking tobacco use, 1990-2021, for the 10 ASEAN countries

Number, share, and percent change in number and share of all-age NCD-associated DALYs attributable to smoking tobacco use, 1990-2021, by location

Location	Number of Smoking Attributable NCD DALYs, 2021 (thousands)	Percent Change in Number of Smoking Attributable NCD DALYs, 1990-2021	Absolute Change in Number of Smoking Attributable NCD DALYs, 1990-2021 (thousands)	Share of NCD DALYs Attributable to Smoking, 2021 (percent)	Percent Change in Share of NCD DALYs Attributable to Smoking, 1990-2021
ASEAN region	13800 (11400, 16200)	96·2 (72·9, 122)	6750 (5070, 8660)	11·2 (9·37, 13·2)	-12·7 (-18·7, -5·20)
Brunei Darussalam	6·22 (4·80, 7·92)	49·0 (25·4, 76·4)	2·04 (1·04, 3·20)	9·61 (7·41, 11·8)	-37·3 (-44·2, -29·1)
Cambodia	321 (242, 399)	109 (62·4, 162)	166 (96·2, 237)	11·9 (9·70, 14·3)	-5·27 (-16·0, 7·91)
Indonesia	6100 (4700, 7710)	171 (111, 237)	3840 (2520, 5350)	11·7 (9·50, 14·1)	14·8 (-2·23, 33·0)
Lao People's Democratic Republic	138 (103, 175)	43·8 (7·88, 86·8)	41·1 (7·90, 75·1)	12·8 (10·4, 15·0)	-13·4 (-23·6, -3·31)
Malaysia	487 (406, 572)	99·1 (83·2, 118)	242 (198, 294)	9·32 (7·69, 11·2)	-22·9 (-28·7, -15·8)
Myanmar	1110 (852, 1420)	-13·6 (-34·4, 13·5)	-188 (-537, 151)	10·2 (8·35, 12·4)	-42·2 (-48·7, -34·7)
Philippines	1970 (1540, 2440)	104 (65·6, 147)	1000 (633, 1440)	11·5 (9·13, 13·7)	-25·2 (-33·4, -15·6)
Singapore	49·2 (38·3, 60·8)	-0·99 (-10·8, 9·03)	-0·47 (-5·30, 4·72)	6·15 (4·73, 7·64)	-49·0 (-53·7, -44·3)
Thailand	1370 (1010, 1840)	45·3 (10·8, 84·4)	425 (114, 794)	8·93 (7·08, 10·9)	-34·5 (-42·7, -25·6)
Viet Nam	2240 (1710, 2720)	123 (71·5, 182)	1220 (765, 1680)	12·7 (10·4, 14·8)	-3·36 (-12·9, 10·1)

Table SM10 Number of smoking attributable deaths for the top five causes of death among people aged 30+, in 2021, for the 10 ASEAN countries

Number of smoking attributable deaths (thousands) for all causes and the top five causes of death among people aged 30+, 2021, by location					
Location	Rank 1 (thousands)	Rank 2 (thousands)	Rank 3 (thousands)	Rank 4 (thousands)	Rank 5 (thousands)
ASEAN Region	IHD	Stroke	COPD	Lung Cancer	Tuberculosis
	118 (99·3, 139)	113 (93·7, 133)	89·3 (72·1, 107)	73·1 (60·8, 86·3)	34·9 (26·7, 44·3)
Brunei Darussalam	IHD	Lung Cancer	COPD	Stroke	Diabetes
	0·046 (0·036, 0·058)	0·043 (0·033, 0·053)	0·024 (0·017, 0·031)	0·019 (0·015, 0·025)	0·010 (0·007, 0·013)
Cambodia	Stroke	IHD	Lung Cancer	COPD	Tuberculosis
	2·63 (1·89, 3·42)	2·53 (1·84, 3·28)	1·98 (1·40, 2·63)	1·88 (1·39, 2·36)	1·69 (0·99, 2·85)
Indonesia	IHD	Stroke	COPD	Lung Cancer	Tuberculosis
	58·6 (44·7, 74·2)	53·8 (41·4, 68·5)	35·4 (26·6, 44·2)	28·1 (19·1, 37·8)	18·9 (13·7, 25·8)
Lao People's Democratic Republic	IHD	Stroke	COPD	Lung Cancer	Tuberculosis
	1·42 (1·06, 1·84)	1·03 (0·75, 1·35)	0·91 (0·65, 1·18)	0·60 (0·43, 0·82)	0·35 (0·15, 0·54)
Malaysia	IHD	Lung Cancer	COPD	LRI	Stroke
	6·08 (5·12, 7·13)	2·71 (2·24, 3·16)	2·63 (2·02, 3·21)	2·33 (1·73, 2·98)	2·26 (1·84, 2·70)
Myanmar	COPD	Stroke	IHD	Lung Cancer	Tuberculosis
	15·4 (11·5, 20·1)	8·66 (6·25, 11·4)	7·79 (5·84, 9·94)	4·65 (3·30, 6·05)	2·47 (1·62, 3·73)
Philippines	IHD	Stroke	COPD	Lung Cancer	LRI
	21·5 (16·8, 26·6)	11·8 (9·19, 14·6)	9·77 (7·41, 12·4)	7·82 (6·19, 9·69)	6·28 (4·58, 8·19)
Singapore	Lung Cancer	IHD	LRI	COPD	Stroke

	0·584 (0·496, 0·688)	0·403 (0·333, 0·488)	0·187 (0·134, 0·251)	0·129 (0·094, 0·167)	0·069 (0·056, 0·086)
Thailand	Lung Cancer	COPD	Stroke	IHD	LRI
	14·0 (10·6, 18·1)	9·11 (6·55, 12·5)	7·55 (5·49, 10·1)	7·54 (5·51, 10·1)	4·67 (3·21, 6·68)
Viet Nam	Stroke	COPD	IHD	Lung Cancer	Tuberculosis
	25·1 (19·3, 31·4)	14·0 (10·5, 18·1)	12·6 (9·37, 15·9)	12·5 (9·57, 15·8)	4·14 (2·88, 6·33)

Table SM11 Smoking prevalence and epidemic phase for ASEAN countries and the corresponding MPOWER implementation statuses

					M	P	O	W		E	R	
**Country**	**Proportion of current female smokers (% and 95% UI)**	**Epidemic phase: Female**	**Proportion of current male smokers (% and 95% UI)**	**Epidemic phase: Male**	**Monitoring smoking**	**Smoking bans**	**Cessation programs**	**Health warnings**	**Mass media**	**Advertising bans**	**Taxation (% of retail price)**	**Cigarettes less affordable since 2012**
**Brunei Darussalam**	5·05 (3·94, 6·39)	2	27·1 (24·4-29·6)	3	⭐⭐⭐	⭐⭐⭐	⭐⭐	⭐⭐⭐	⭐⭐	⭐⭐	No data	No data
**Cambodia**	5·53 (4·53, 6·67)	2	40·5 (38·6-42·6)	3	⭐⭐⭐	⭐⭐⭐	⭐⭐	⭐⭐⭐	⭐⭐	⭐⭐	26·4	❌
**Indonesia**	3·54 (2·92, 4·36)	1	57·8 (56·2-59·4)	2	⭐⭐⭐	⭐	⭐⭐	⭐⭐	❌	❌	72·9	↔️
**Lao People's Democratic Republic**	6·82 (5·64, 8·28)	2	48·6 (46·1-51·1)	3	⭐⭐⭐	⭐⭐⭐	❌	⭐⭐⭐	❌	⭐⭐⭐	15·4	❌
**Malaysia**	3·06 (2·46, 3·80)	1	39·9 (37·3-42·4)	3	⭐⭐⭐	❌	⭐⭐	⭐⭐⭐	⭐⭐⭐	⭐⭐	51·6	↔️
**Myanmar**	8·01 (6·63, 9·46)	4	39·6 (37·6-41·8)	3	❌	⭐	⭐⭐	⭐⭐⭐	❌	⭐⭐	36·0	↔️
**Philippines**	7·91 (6·76, 9·25)	4	39·9 (37·9-41·9)	3	⭐⭐⭐	⭐	⭐⭐⭐	⭐⭐⭐	⭐⭐	⭐⭐	50·6	✅
**Singapore**	6·56 (5·20, 8·19)	4	20·2 (18·4-22·3)	4	⭐⭐⭐	⭐	⭐⭐⭐	⭐⭐⭐	⭐	⭐⭐	66·3	❌
**Thailand**	3·36 (2·70, 4·09)	1	39·9 (38·0-41·8)	3	⭐⭐⭐	⭐⭐⭐	⭐⭐	⭐⭐⭐	⭐⭐⭐	⭐⭐	81·3	↔️

**Viet Nam**	2·57 (2·04, 3·20)	1	46·9 (45·0-48·9)	3	★★★	★★	★★	★★★	★★★	★★	34·3	✗

★ = level of implementation, where more stars indicate more complete implementation
✗ = no implementation/cigarettes more affordable since 2012
↔ = cigarette affordability no different from 2012
✓ = cigarettes less affordable since 2012

GATHER Checklist

Item #	Checklist item	Reporting location
Objectives and funding		
1	Define the indicator(s), populations (including age, sex, and geographic entities), and time period(s) for which estimates were made.	Main text methods overview, paragraph 1
2	List the funding sources for the work.	Main text method section "role of the funders"
Data Inputs		
For all data inputs from multiple sources that are synthesized as part of the study:		
3	Describe how the data were identified and how the data were accessed.	Main text methods section "Prevalence of smoking": paragraph 1; "Dose-response risk curves": paragraph 2 "
4	Specify the inclusion and exclusion criteria. Identify all ad-hoc exclusions.	Exclusion criteria summarized in Methods section section "Prevalence of smoking": paragraph 1; "Dose-response risk curves" :paragraph 2; full inclusion and exclusion criteria listed in SM, section "PRISMA Flowcharts"
5	Provide information on all included data sources and their main characteristics. For each data source used, report reference information or contact name/institution, population represented, data collection method, year(s) of data collection, sex and age range, diagnostic criteria or measurement method, and sample size, as relevant.	Table SM1 "List of data sources included to estimate current smoking prevalence from the 10 ASEAN countries"; citations also given on the GHDx (https://ghdx.healthdata.org/);
6	Identify and describe any categories of input data that have potentially important biases (e.g., based on characteristics listed in item 5).	Data inputs in excel format available on the GHDx (https://ghdx.healthdata.org/)
For data inputs that contribute to the analysis but were not synthesized as part of the study:		
7	Describe and give sources for any other data inputs.	N/A
For all data inputs:		
8	Provide all data inputs in a file format from which data can be efficiently extracted (e.g., a spreadsheet rather than a PDF), including all relevant meta-data listed in item 5. For any data inputs that cannot be shared because of ethical or legal reasons, such as third-party ownership, provide a contact name or the name of the institution that retains the right to the data.	Data inputs in excel format available on the GHDx (https://ghdx.healthdata.org/)
Data analysis		
9	Provide a conceptual overview of the data analysis method. A diagram may be helpful.	Main text methods overview; Model flow chart in the supplementary materials
10	Provide a detailed description of all steps of the analysis, including mathematical formulae. This description should cover, as relevant, data cleaning, data pre-processing, data adjustments and weighting of data sources, and mathematical or statistical model(s).	Main text methods; supplementary materials from the section "model flowchart" to "PAF calculation"
11	Describe how candidate models were evaluated and how the final model(s) were selected.	Main text methods; supplementary materials from the section "model flowchart" to "PAF calculation"
12	Provide the results of an evaluation of model performance, if done, as well as the results of any relevant sensitivity analysis.	N/A

13	Describe methods for calculating uncertainty of the estimates. State which sources of uncertainty were, and were not, accounted for in the uncertainty analysis.	Main text methods section "Prevalence of smoking" paragraph 2 and section "Dose-response risk curves" paragraph 1
14	State how analytic or statistical source code used to generate estimates can be accessed.	GitHub URL will be provided at resubmission
Results and Discussion		
15	Provide published estimates in a file format from which data can be efficiently extracted.	The results can be efficiently extracted at https://vizhub.healthdata.org/gbd-results/
16	Report a quantitative measure of the uncertainty of the estimates (e.g. uncertainty intervals).	UIs given for all findings, including in the text, figures, and tables in the main text and SM; online viz tools (see information above)
17	Interpret results in light of existing evidence. If updating a previous set of estimates, describe the reasons for changes in estimates.	Main text discussion paragraphs 1-11
18	Discuss limitations of the estimates. Include a discussion of any modelling assumptions or data limitations that affect interpretation of the estimates.	Main text discussion paragraph 12

PRISMA Flowcharts

Prisma flow charts can be found in previous publication.

Author Contributions

Managing the overall research enterprise

Xiaochen Dai, Emmanuela Gakidou, Simon I Hay

Writing the first draft of the manuscript

Xiaochen Dai and Marie Ng

Primary responsibility for applying analytical methods to produce estimates

Xiaochen Dai, Gabriela Gil, Brooks W Morgan

Primary responsibility for seeking, cataloguing, extracting, or cleaning data; designing or coding figures and tables

Xiaochen Dai, Gabriela Gil, Brooks W Morgan, Jason A Anderson

Providing data or critical feedback on data sources

Qorinah Estiningtyas Sakilah Adnani, Budi Aji, Syed Mohamed Aljunid, Gianna Gayle Herrera Amul, Jason A Anderson, Sumadi Lukman Anwar, Geminn Louis Carace Apostol, Sarunya Benjakul, Bryan Chong, Dinh-Toi Chu, Xiaochen Dai, Thanh Chi Do, Diyan Ermawan Effendi, Gabriela Fernanda Gil, Arief Hargono, Eka Mishbahatul Marah Has, Hong-Han Huynh, Dian Kusuma, Tri Laksono, Nhi Huu Hanh Le, Thao Thi Thu Le, Stefan Ma, Roy Rillera Marzo, Mustapha Mohammed, Brooks W Morgan, Christopher J L Murray, Phat Tuan Nguyen, Van Thanh Nguyen, Sok King Ong, Sher Zaman Safi, Made Ary Sarasmita, Siddharthan Selvaraj, Sunil Shrestha, Chandrashekhar T Sreeramareddy, Narayanaswamy Venketasubramanian, Maniphanh Vongphosy, Tati Suryati Suryati Warouw, Angga Wilandika, Siti Rosemawati Yussof

Developing methods or computational machinery

Xiaochen Dai, Emmanuela Gakidou, Gabriela Fernanda Gil, Simon I Hay, Christopher J L Murray

Providing critical feedback on methods or results

Qorinah Estiningtyas Sakilah Adnani, Budi Aji, Syed Mohamed Aljunid, Jason A Anderson, Sumadi Lukman Anwar, Geminn Louis Carace Apostol, Kurnia Dwi Artanti, Sarunya Benjakul, Amiel Nazer C Bermudez, Bryan Chong, Dinh-Toi Chu, Xiaochen Dai, Thanh Chi Do, Ferry Efendi, Diyan Ermawan Effendi, Emmanuela Gakidou, Gabriela Fernanda Gil, Eka Mishbahatul Marah Has, Simon I Hay, Hong-Han Huynh, Endang Indriasih, Muhammad Iqhrammullah, Ammar Abdulrahman Jairoun, Kehinde Kazeem Kanmodi, Helda Khusun, Maria Dyah Kurniasari, Dian Kusuma, Tri Laksono, Nhi Huu Hanh Le, Thao Thi Thu Le, Stefan Ma, Roy Rillera Marzo, Mustapha Mohammed, Brooks W Morgan, Christopher J L Murray, Marie Ng, Phat Tuan Nguyen, Van Thanh Nguyen, Dina Nur Anggraini Ningrum, Efaq Ali Noman, Sok King Ong, Sher Zaman Safi, Siddharthan Selvaraj, Sunil Shrestha, Solikhah Solikhah, Chandrashekhar T Sreeramareddy, Ingan Ukur Tarigan, Jansje Henny Vera Ticoalu, Narayanaswamy Venketasubramanian, Maniphanh Vongphosy, Tati Suryati Suryati Warouw, Angga Wilandika, Siti Rosemawati Yussof

Drafting the work or revising it critically for important intellectual content

Qorinah Estiningtyas Sakilah Adnani, Gianna Gayle Herrera Amul, Geminn Louis Carace Apostol, Kurnia Dwi Artanti, Bryan Chong, Dinh-Toi Chu, Xiaochen Dai, Thanh Chi Do, Diyan Ermawan Effendi, Nelsensius Klau Fauk, Gabriela Fernanda Gil, Arief Hargono, Eka Mishbahatul Marah Has, Simon I Hay, Hong-Han Huynh, Kehinde Kazeem Kanmodi, Maria Dyah Kurniasari, Dian Kusuma, Nhi Huu Hanh Le, Thao Thi Thu

Le, Roy Rillera Marzo, Mustapha Mohammed, Christopher J L Murray, Gustavo G Nascimento, Marie Ng, Phat Tuan Nguyen, Van Thanh Nguyen, Bedanta Roy, Siddharthan Selvaraj, Sunil Shrestha, Solikhah Solikhah, Chandrashekhar T Sreeramareddy, Yen Lian Tan, Thien Tan Tri Tai Truyen, Narayanaswamy Venketasubramanian, Tati Suryati Suryati Warouw, Siti Rosemawati Yussof

Managing the estimation or publications process

Xiaochen Dai, Emmanuela Gakidou, Simon I Hay, Christopher J L Murray

References

1 Stevens GA, Alkema L, Black RE, *et al.* Guidelines for Accurate and Transparent Health Estimates Reporting: the GATHER statement. *The Lancet* 2016; **388**: e19–23.

2 Ng M, Freeman MK, Fleming TD, *et al.* Smoking Prevalence and Cigarette Consumption in 187 Countries, 1980-2012. *JAMA* 2014; **311**: 183–92.

3 Reitsma MB, Kendrick PJ, Ababneh E, *et al.* Spatial, temporal, and demographic patterns in prevalence of smoking tobacco use and attributable disease burden in 204 countries and territories, 1990–2019: a systematic analysis from the Global Burden of Disease Study 2019. *The Lancet* 2021; **397**: 2337–60.

4 Zheng P, Afshin A, Biryukov S, *et al.* The Burden of Proof studies: assessing the evidence of risk. *Nat Med* 2022; **28**: 2038–44.

5 Zheng P, Barber R, Sorensen RJD, Murray CJL, Aravkin AY. Trimmed Constrained Mixed Effects Models: Formulations and Algorithms. *Journal of Computational and Graphical Statistics* 2021; **30**: 544–56.

6 Naghavi M, Ong KL, Aali A, *et al.* Global burden of 288 causes of death and life expectancy decomposition in 204 countries and territories and 811 subnational locations, 1990–2021: a systematic analysis for the Global Burden of Disease Study 2021. *The Lancet* 2024; **0**. DOI:10.1016/S0140-6736(24)00367-2.

7 Ferrari AJ, Santomauro DF, Aali A, *et al.* Global incidence, prevalence, years lived with disability (YLDs), disability-adjusted life-years (DALYs), and healthy life expectancy (HALE) for 371 diseases and injuries in 204 countries and territories and 811 subnational locations, 1990–2021: a systematic analysis for the Global Burden of Disease Study 2021. *The Lancet* 2024; **0**. DOI:10.1016/S0140-6736(24)00757-8.

8 Brauer M, Roth GA, Aravkin AY, *et al.* Global burden and strength of evidence for 88 risk factors in 204 countries and 811 subnational locations, 1990–2021: a systematic analysis for the Global Burden of Disease Study 2021. *The Lancet* 2024; **403**: 2162–203.

9 Dai X, Gil GF, Reitsma MB, *et al.* Health effects associated with smoking: a Burden of Proof study. *Nat Med* 2022; **28**: 2045–55.
